# Crop cover and nutrient levels mediate the effects of land management type on aquatic invertebrate richness in prairie potholes

**DOI:** 10.1371/journal.pone.0295001

**Published:** 2024-04-16

**Authors:** David Anthony Kirk, Sara J. Collins, Juan Andrés Martínez-Lanfranco, Amanda E. Martin

**Affiliations:** 1 Aquila Conservation & Environment Consulting, Carlsbad Springs, Ontario, Canada; 2 Geomatics and Landscape Ecology Laboratory (GLEL), Ottawa-Carleton Institute of Biology, Carleton University, Ottawa, Ontario, Canada; 3 Department of Biological Sciences, University of Alberta, Centennial Centre for Interdisciplinary Science Building, Edmonton, Alberta, Canada; 4 National Wildlife Research Centre, Environment and Climate Change Canada, Ottawa, Ontario, Canada; 5 Department of Biology, Carleton University, Ottawa, Ontario, Canada; CIFRI: Central Inland Fisheries Research Institute, INDIA

## Abstract

Aquatic invertebrates provide important ecosystem services, including decomposition and nutrient cycling, and provide nutrition for birds, fish, amphibians, and bats. Thus, the effects of agricultural land management practices on aquatic invertebrates are relevant to farmers, wildlife biologists, and policymakers. Here, we used data on aquatic invertebrates (159 taxa, 73 to species, 75 to genus/family) collected in 40 wetlands in the Canadian prairies to test for direct and indirect relationships among land management types (perennial cover, organic, minimum tillage, conventional), landscape structure (cropland and wetland cover within the surrounding landscape), and water quality (total nutrient levels, turbidity) on species richness of invertebrates using structural equation modelling. Additionally, we assessed variation in community composition within and among wetlands in different land use management types using a direct gradient analysis and variance partitioning. The direct effects of land management type were not supported but we found strong supportive evidence that effects of land management on richness were significantly mediated through cropland cover, nutrient levels, and turbidity. After controlling for these indirect effects, aquatic invertebrate richness decreased along a gradient from the lowest to the highest farming intensity, i.e., richness decreased from perennial cover sites to organic to minimum tillage to conventional sites. Support was also found for negative effects of nutrient levels and turbidity on richness. We did not find significant support for differences in gamma diversity or a simple test (homogeneity of multivariate dispersions) of differences in turnover among land management types; however, land management had a significant effect in distance-based redundancy analysis. Taken together, these results suggest that focusing conservation efforts on reducing cropland erosion and nutrient inputs to wetlands and creating more permanent cover may be effective strategies for conserving richness of aquatic invertebrates in agricultural landscapes in this region.

## Introduction

As a critical component of wetland biodiversity, aquatic invertebrates contribute to both wetland function and provision of ecosystem services [1, 2]. They act as a vital link between primary production and secondary consumers [3], providing a crucial dietary element for birds, fish, amphibians, and bats [4–6]. For instance, aquatic invertebrates supply waterfowl with nutrition for egg-laying, as well as easily-captured larvae for developing ducklings and molting birds [7]. Moreover, aquatic invertebrates play a pivotal role in aquatic ecosystem functioning and deliver important supporting ecosystem services through their influence on nutrient cycles, primary productivity, decomposition and organic matter transfer [8–13]. In addition, wetland-associated invertebrates provide a food source for aerial avian insectivores which consume cropland pest species [14], potentially benefiting agriculture through their predatory ecosystem services [15].

Here, we focus on the effects of three agricultural land management types (management for perennial cover, organic, minimum tillage farming) that are generally expected to have reduced impacts on biodiversity relative to conventional farming. Such management practices in agricultural landscapes are often implemented to reduce soil erosion in upland sites, reduce sediment and nutrient loadings, and improve water quality in wetlands to the mutual advantage of both farmers (in terms of ecosystem services) and biodiversity [16–18]. However, it is not clear how factors associated with different land management types influence aquatic invertebrates [4, 19–22], and the influence of land management types on aquatic invertebrates has only been well-studied in a few regions [20].

Management for perennial cover largely involves conversion of cultivated land to grazed, annual pasture or hayed lands [23]. In the North Dakota prairie potholes, researchers found higher richness and abundance of cladoceran resting eggs (ephippa), planorbid and physid snail shells, and ostracod shells in wetlands surrounded by grasslands than those surrounded by croplands [24]. Other studies similarly suggest that conversion to perennial cover in landscapes surrounding water bodies would be beneficial for aquatic invertebrates [25–27], probably largely because erosion and topsoil deposition from cropland can reduce wetland storage volume and cause sedimentation [16]. However, the effects of two programs that promote perennial cover—the United States’ Conservation Reserve Program (CRP) and the affiliated Conservation Reserve Enhancement Program (CREP)—on stream aquatic invertebrates are equivocal. For example, South et al. [28] found that the number of first order tributaries, soil permeability and urban land had a far greater effect on stream invertebrate communities than landscape level CRP/CREP lands. Nevertheless, the relationship between perennial cover and decreased nutrient loads is documented in other systems, such as tallgrass prairie [29, 30], and would be expected to have a positive effect on aquatic invertebrates.

Minimum tillage involves retaining at least 30% of crop residue from the previous crop following seeding [31]. This practice reduces soil erosion and thus nutrient loss [32]. It also results in lower sediment levels in aquatic systems, and sedimentation has been shown to have a negative effect on emergence of aquatic invertebrates [33]. Some research indicates that runoff from agricultural land influences aquatic invertebrate assemblages via increasing turbidity [18, 34]. Minimum tillage is also associated with increased use of herbicides to combat weed incursion [35], but there are few studies of the effects of minimum tillage on aquatic invertebrates and these mostly involve stream ecosystems (e.g., [36]).

Organic farming embraces a holistic approach to agriculture [37, 38]. A key distinguishing feature of organic farming is its avoidance of synthetic fertilizers and pesticides. In New Zealand, organic farming and integrated farming systems benefitted stream aquatic invertebrates compared to conventional farming [39]. Conventional farms had consistently lower species richness as well as richness of Ephemeroptera, Plecoptera and Tricoptera and a lower Macroinvertebrate Community Index (an index that weights species’ presence/absence in terms of their organic pollutants tolerance). Compared to organic and integrated farming systems, conventional farms also had less representation of species and conventional farms also had significant changes in trait composition (e.g., body size, dietary preference). In Brazil, functional composition of aquatic invertebrates varied between organic and conventional rice fields, with more species having predatory traits and more pesticide-sensitive genera in organic fields [40]; however, taxonomic richness and abundance were similar between organic and conventional rice fields [41].

Lastly, conventional farming is characterized by its use of synthetic pesticides and fertilizers, and increased tillage (> 70% tilled) which leads to increased sediment runoff [24]. Aquatic invertebrates are sensitive to insecticides ([42, 43]; especially neonicotinoids and pyrethroids) and trends in the total applied toxicity (the reciprocal of group-specific regulatory thresholds for non-target species multiplied by annual applications of pesticides [44]) susceptibility rating of aquatic invertebrates have been increasing since 1992 [44]. Moreover, a recent meta-analysis demonstrated that nitrogen (N) and phosphorus (P)—both of which are typically used liberally on conventional farms—generally had negative effects on terrestrial and aquatic invertebrates [45]. However, despite the negative effects of agriculture on aquatic systems, wetlands in conventional farmland can also provide important aquatic habitat for wildlife species in regions that have lost much of their historical wetland cover [46–48].

Beyond effects of different land management types on aquatic invertebrate communities, it is possible that agricultural operations and conversion of natural/semi-natural to agricultural land cover has affected aquatic invertebrate communities [49]. Agricultural operations have resulted in drainage of wetlands since Euro-American settlement, amounting to between 60–65% loss of historical wetlands since 1900 in the Prairie Pothole Region (PPR; [50]). This historical loss and fragmentation of wetlands has resulted in direct habitat loss for aquatic invertebrates, as well as increased distances between wetlands leading to decreased dispersal and subsequent population declines [24]. Agriculture has also resulted in the loss of ~75% of native mixed-grass prairie in the Northern Great Plains [51], largely due to conversion to cropland [52].

Conversion to cropland removes land cover such as native grassland, as well as other natural and semi-natural land cover features (e.g., woodland groves), which could have negative impacts on aquatic invertebrate communities. It can directly impact overall water quality in prairie wetlands since these may act as filtration systems moderating and reducing runoff and sedimentation [18, 24]. Increases in sedimentation from erosion due to expanding crop cover [18] has also probably led to decreases in aquatic invertebrate richness and diversity in wetlands surrounded by cropland, as well as potentially invertebrate egg banks [17, 24, 33, 49]. Conversion of native grassland and woodland to agriculture can also result in a loss of terrestrial habitat for the adult life stages of some aquatic invertebrate species [53].

Increasing wetland cover within the landscape surrounding wetlands (‘potholes’) has been shown to benefit invertebrate communities in the PPR [54], most likely because this is related to habitat amount for invertebrate species and opportunities for colonization, which also depends on wetland heterogeneity and connectivity/spatial organization [55]. In addition to those benefits, we also expected that increasing wetland cover in the landscape would improve water quality (i.e., lower nutrient levels and turbidity) since many studies show a link between wetlands, even geographically isolated ones, and landscape function such as nutrient, sediment and floodwater retention [56, 57].

Evidence also suggests that in landscapes dominated by cropland, nutrient inputs to nearby wetlands and wetland turbidity may increase, sometimes causing negative effects on aquatic invertebrates. For example, Collins et al. [58] found that the proportional cover of cropland had strong, negative effects on invertebrate richness in farm drainage ditches, mediated by the relationship between crop cover and the levels of N and dissolved oxygen in ditches. Turbidity has been shown to have negative impacts on aquatic invertebrates via its direct physical effects via toxicity (e.g., ingestion of sediment particles), as well as indirect effects via reduced food supply (e.g., phytoplankton), burial of invertebrate egg banks potentially resulting in reduced emergence [33], or interfering with interstitial spaces used as habitat by species; conversely, positive indirect effects include refuge from fish predation provided by increased turbidity [59]. Note that this study focused on total suspended solids which is usually positively correlated with turbidity.

**Fig 1:**
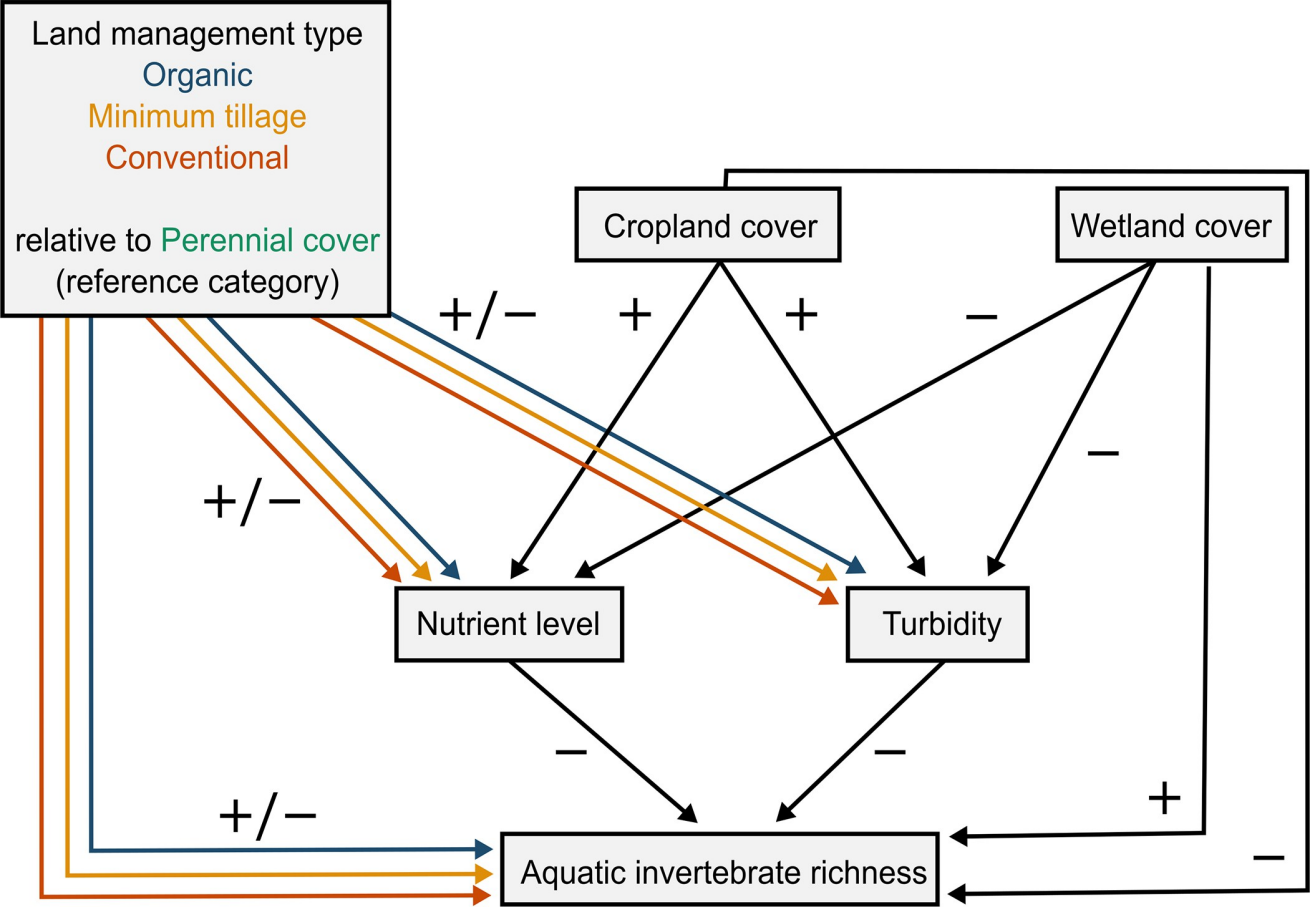
Predicted responses of aquatic invertebrate species richness as a function of land management types (organic, minimum tillage, and conventional relative to perennial cover), water quality (nutrient level and turbidity), and landscape structure (cropland and wetland cover). The expected relationships are represented as arrows originating from hypothesized drivers pointing to response variables, where the direction of effects are shown as positive (+), negative (–), or mixed (+/–).

Here we tested hypothesized direct and indirect relationships among land management types, landscape structure (cropland and wetland cover), nutrient levels, turbidity, and aquatic invertebrate richness using data collected at 40 wetlands in Saskatchewan, Canada. We define a direct effect as the effect from one predictor directly on aquatic invertebrate richness (e.g., effect of land management on richness derived from a model of richness as a function of land management type) and an indirect effect as the effect of one predictor on richness via an intermediate predictor (e.g., effect of land management on richness, from a combination of models of richness as a function of nutrient level and nutrient level as a function of land management type). Our predictions are shown in Fig 1.

To summarize: we expected wetland total nutrient levels (N + P) and turbidity to have negative effects on aquatic invertebrates. We expected both of these variables would be influenced by land management type and landscape structure; however, we did not have a specific prediction regarding the direction of effects (i.e., positive or negative) of land management type on nutrients. Nutrient levels do not necessarily increase with agricultural intensification. For example, applications of inorganic fertilizer on conventional farms may lead to elevated nutrient levels, but applications of manure fertilizer on organic farms could also increase nutrient levels in adjacent wetlands, as could livestock grazing on perennial cover sites. Similarly, we did not have a specific directional prediction for the possible effects of land management type on turbidity. However, we did expect higher nutrient levels and turbidity in wetlands surrounded by more cropland and less wetland. Thus, we expected indirect, negative effects of cropland cover and positive indirect effects of wetland cover on aquatic invertebrates. We also expected a direct effect of land management on aquatic invertebrates, with greater richness associated with organic and perennial sites relative to minimum tillage and conventional, because of their higher quality terrestrial vegetation via greater diversity of terrestrial plant cover types, and lack of synthetic pesticide use. Finally, we expected direct effects of cropland and wetland cover; increasing cropland cover results in a reduction of available terrestrial land cover types used by species as habitat within the landscape while increasing wetland cover increases the total amount of aquatic cover types.

As a complement to the path analysis, we analyzed differences in regional gamma diversity (γ) and beta diversity (β) among land management types, in this case expecting sites with lower management intensity (i.e., perennial cover, organic) to have higher estimated γ and β than more intensively managed systems (i.e., conventional, minimum tillage). Our predictions differed somewhat from the path analysis; although they were similar in that we had expectations based on direct effects (i.e., land management types), they differed in that we could not examine indirect effects of other factors via land management type (or other paths). Instead, ordination axes produced linear combinations of environmental variables. Finally, we used Indicator Species Analysis [60] to identify species associated with specific land management types.

## Materials and methods

### Study area and site selection

**Fig 2:**
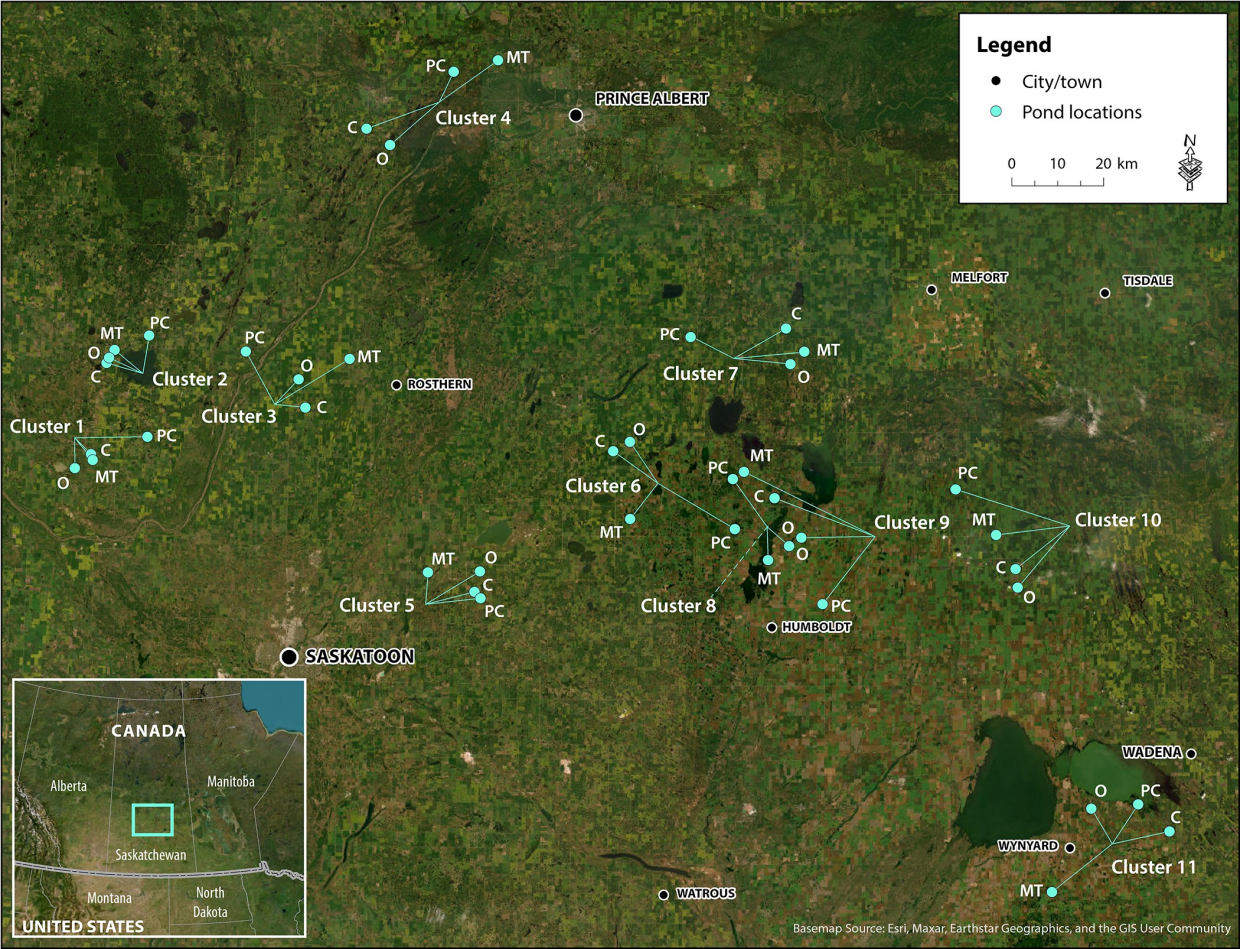
Map showing positions of 11 clusters of farms and their associated wetlands (sites), with four sites per cluster. Each cluster includes a wetland for each land management type (PC = perennial cover; O = organic; MT = minimum tillage; C = conventional), near Saskatoon, Saskatchewan, Canada. “Background imagery reprinted from [71] under a CC BY license, with permission from [ESRI], original copyright [2015]”.

Our study sites were located within the Aspen Parkland and Boreal Transition ecoregions in the PPR of south-central Saskatchewan, Canada ([61]; Fig 2). The PPR encompasses thousands of small depressions formed by the Wisconsin glaciation which collectively form a globally important wetland complex [62, 63], providing habitat for millions of breeding migratory waterfowl species that contribute to provisioning, regulating and cultural services [64–66]. These small, shallow wetland systems (prairie ‘potholes’ in the United States, or prairie ‘sloughs’ in Canada) are referred to as ‘wetlands’ hereafter. Despite the importance of the PPR for biodiversity and other ecosystem services, it has been subjected to extensive landscape modification, largely from agriculture [67]. Compared to other ecoregions in the Prairie Parkland Region of Canada, the Aspen Parkland, and the Boreal Transition ecoregions, incurred the greatest loss of wetlands between 1985 and 2001 [68].

This study was set up by Agriculture and Agri-Foods Canada (AAFC; [69]), and Environment and Climate Change Canada (ECCC; [70]). A total of 44 farms was initially selected, using the following approach. First, 11 clusters of farms were identified, where each cluster included four farms: one perennial cover, one organic, one minimum tillage, and one conventional. For each cluster, the organic farm was identified first by AAFC, selecting a site (farm) in agricultural production and associated pond within a certified organic farm, where no synthetic agrochemicals had been used for a minimum of four years. Many, if not most, organic farms had never experienced synthetic pesticide use and were usually owned by the same farming family over several generations.

Organic farm sites were selected first because this land management type was rare within the study area [72]. Each organic farm site was matched with a perennial cover, minimum tillage and conventional site, selected from the candidate sites within a 25 km radius of the organic site. This resulted in 11 ‘clusters’ of sampling sites, where each cluster contained one site within each land management category (Fig 2). This clustering was used to try and ensure that all land management types within a cluster would have similar soil types, topography and climate; other criteria (e.g., farm acreage) could also have been used to further constrain site selection and increase the similarity of sites within a cluster. Perennial cover sites were on uncultivated land owned or leased by the provincial government, federal government or Ducks Unlimited Canada, or managed lands that were part of the Permanent Cover Program (PCP). Two-thirds (6 of 11) of the perennial cover sites were comprised of native grassland (i.e., substantial areas of native grassland in the wetland catchment), while one site had some native grassland but the catchment was mostly aspen (*Populus tremuloides*) forest. These were managed to provide habitat for wildlife species. Three perennial cover sites were known to be grazed (cattle present on PCP sites at Clusters 2, 5 and 11; Fig 2), but other sites could also have possibly been grazed. No pesticides were applied at perennial cover sites. Minimum tillage sites were tilled less than three times a year and treated with pesticides two or more times per year. On conventional sites, farmers tilled their soil more than three times a year and typically applied more than one pesticide treatment each year [72]. Organic farmers also used tillage for weed control and may have tilled their fields more frequently than farmers on other land management types.

Second, ECCC field crews re-evaluated the candidate wetland sites within ‘quarter sections’ of each of the farms that contained at least one wetland with its associated drainage basin (determined from topographic maps) restricted to that quarter section. A quarter section is ~65 ha or ¼ square mile and a subdivision of a 260 ha section or one square mile, which is a land unit used in land surveys. This process involved re-selection of many farms because they lacked suitable wetlands for study (D. Donald, personal communication). Note that about half of these selected farm and associated wetland sites overlapped with focal sampling sites for plants, terrestrial invertebrates, and birds as separate projects that were part of the larger study [70, 72, 73]. Many prairie wetlands undergo seasonal fluctuations in water levels, experiencing substantial inputs from snowmelt in the spring, followed by periods of drying in the late summer [74]. All selected wetlands were wet during the invertebrate sampling period (over the entire period of May to early July) and had only a single land management type within the wetland drainage basin, to reduce potential runoff of contaminants from other land use types. Because salinity and water depth (pond permanence) are known to influence aquatic invertebrate communities [75, 76], wetlands were selected with the following characteristics: low total dissolved solids (TDS < 1000 mg/L), a May-July depth of about 1 m (± SD 0.5 m), an open water area of about 2 ha and at least some tall vegetation lining their shores (D. Donald, personal communication). We note that, although turbidity can sometimes be strongly related to TDS, in fact this was not the case in our study (R = -0.166, P = 0.305).

### Aquatic invertebrate survey

Due to logistical constraints, aquatic invertebrates were surveyed at only 40 of the 44 selected sites. Each wetland was visited twice in 1996: once in May and again between late June and early July. In May, anostracans were collected using 10 standardized dip-net (mesh size 0.8 × 0.9 mm) samples filtered through a 153 μm^2^ mesh net. In late June–early July, invertebrates were sampled with 10 dip–net sweeps and zooplankton (including calanoids) were collected by filtering 16–48 litres of water through a 153 μm^2^ mesh net.

All zones within each wetland were sampled, including open water, near shore, and emergent and submergent vegetation. For logistical reasons, the 3–4 sites within a cluster were sampled consecutively, over a period of several days. The order of sampling within and among clusters depended on the timing of herbicide applications. Invertebrates were collected in the minimum tillage and conventional wetlands only after farmers confirmed that herbicide applications in the basin had been completed. Likewise, sampling of perennial cover and organic sites within a cluster was delayed until herbicide applications were complete for the matching minimum tillage and/or conventional sites.

The following process was used to identify and estimate relative abundances of different invertebrate taxa from both the May and June–July dip-net samples. For relatively small samples (up to ~500 individuals), all invertebrates were sorted from each dip-net sample and identified to the lowest taxonomic level possible (typically species; otherwise, to genus or family), recording the percent of the sample sorted. For larger samples (≥ 500 individuals), either 50% or 25% of the sample was sorted, and we adjusted abundance to 100% to standardize the abundance estimate, by multiplying the count by 2 or 4, respectively. In the case of adult Corixidae, all specimens were sorted because a high diversity of corixids was expected, and these can be easily identified at the adult stage. For the remaining portion of a sample (if any), only taxa/genera not represented in the initial collection were identified to species/genus/family and recorded as ‘rare’. We note that the genera/family level identifications were treated as ‘species’ in the analyses (a list of all taxa identified is in S1 Table).

### Environmental data

At each wetland, nutrients, dissolved ions, turbidity and physical characteristics such as adjacent vegetation were assessed (water quality variables are in S2 Table). Water quality variables were measured using standardized methods [77] taking one water sample (using separate 1 L bottles for nutrients, ammonia and ions) from each pond between late June and early July. We used the total nutrients (the sum of total P and total N) and turbidity in our analyses (see next section) as these water quality variables are known to be important for aquatic invertebrates and can be influenced by land management type and surrounding landscape structure. Turbidity was measured using a nephelometer. We combined N and P into a single measure to reduce the number of variables in the statistical models.

We also calculated the proportional cover of cropland and wetlands (pooling the area of water bodies, marsh and mud/sand/saline) within a 1-km radius of each wetland (a GPS location taken at each wetland edge). Note that for cropland we included only crops such as canola and spring/winter wheat and did not include hay (see S3 Table), although technically hay is a crop. We treated hay separately because it has lower pesticide applications than row and field crops. The 1-km radius is consistent with the landscape extent used in a recent study of the effects of landscape variables on invertebrates in agricultural landscapes [58]. We used data from a published land cover classification map derived from a Landsat imagery for the early–mid 1990s ([78]; S3 Table**)**. In addition, for each wetland, local-scale variables were collected such as open water area, total wetland area and pond depth in the early (May) and late (July) season (S1 Fig).

### Statistical analyses

#### Direct and indirect effects of land management type, landscape structure, and water quality on aquatic invertebrates

We used confirmatory path analysis (structural equation modelling) to simultaneously model all hypothesized direct and indirect relationships among the predictor variables and aquatic invertebrate species richness (Fig 1). This analysis uses the directional separation (d-sep) test [79] and readily accommodates small to moderate sample sizes, which was appropriate for our study. The farm clusters were used as a random effect in the models to account for spatial patterns. Prior to analysis, we standardized all water quality and landscape variables to a mean of 0 and standard deviation (SD) of 1. ‘Land management type’ was a categorical variable, and the coefficients for organic, minimum tillage and conventional land management types in the path analysis represented the average difference in a response between these management types and the perennial cover management type (i.e., the reference category). Thus, the coefficients for the three land management types present in the model are relative to a reference condition (perennial cover).

**Fig 3:**
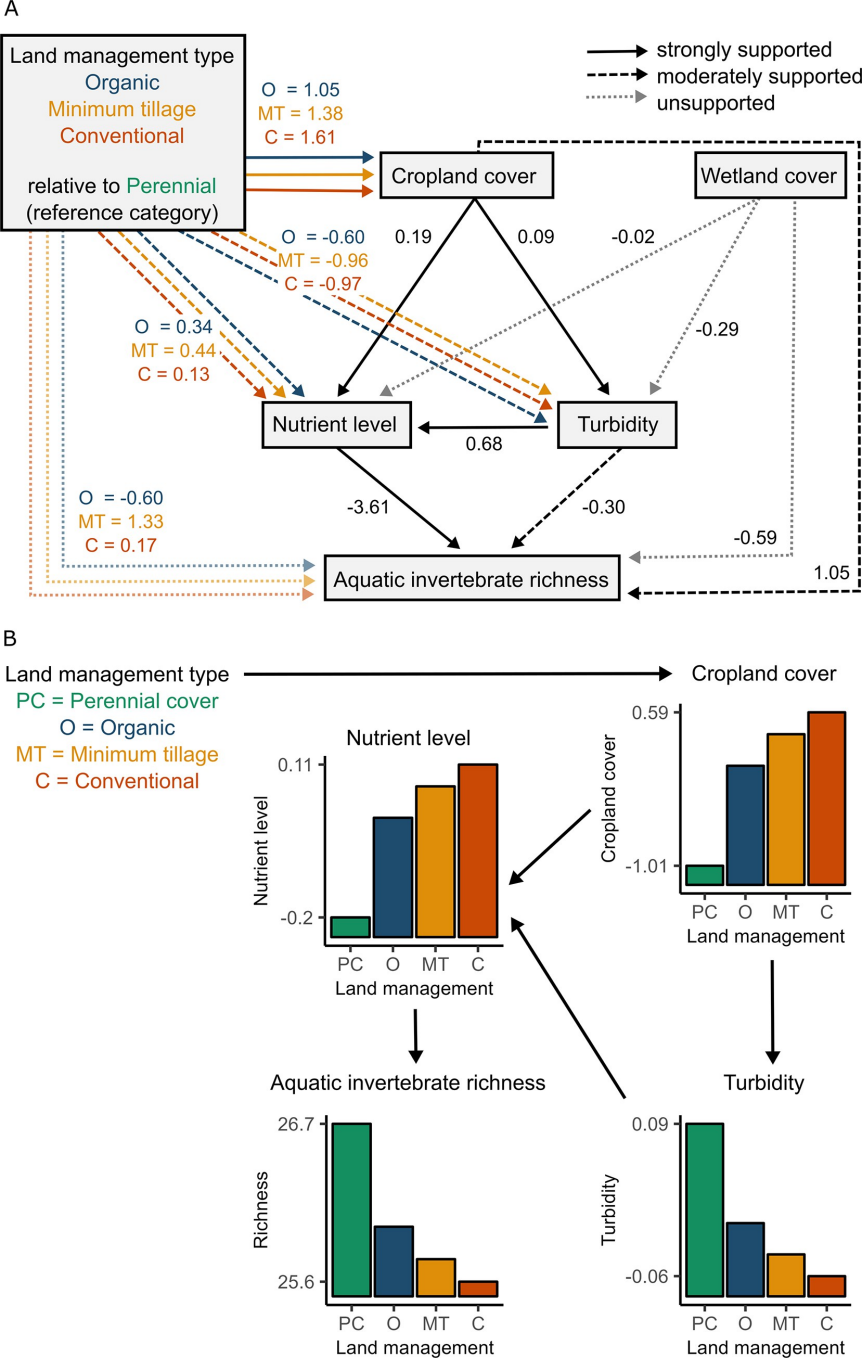
a) Path model estimates for relationships among land management type, water quality (standardized total nutrient levels and turbidity), landscape structure (standardized cropland and wetland cover), and aquatic invertebrate richness (i.e., observed richness) from the full (global) model. Coefficients for organic, minimum tillage and conventional land management types represent the average difference in the response between that type and perennial cover (i.e., used as reference). Paths are considered strongly supported if they are included in the most supported model (based on Akaike Information Criterion [AIC]) and paths included in models within 2 AIC of the most supported model are moderately supported. Analytical results are in S5 Table. All responses were modeled using linear mixed models with cluster as a random factor. b) Predicted direct and indirect effects of land management type on cropland cover, nutrient levels, turbidity and aquatic invertebrate richness, from the most supported candidate path model (solid black arrows in a). To model the indirect effects of land management type on richness (via cropland cover, nutrient levels and turbidity), we first predicted the relative cropland cover for each land management type, and then used these four cropland cover values to predict turbidity. We used these predictions for cropland cover and turbidity to predict the nutrient levels for each land management type, and finally used the four nutrient level values to predict species richness.

To evaluate the correlational structure of our hypothesized path model we used the d-sep test before parameterizing individual paths between variables [79, 80]. This test involved testing each implied independency (i.e., every pair of variables predicted to lack a direct relationship) in the path model using a linear mixed model design to determine the probability that each pair was statistically independent (conditional on the hypothesized predictors in Fig 1). The correlational structure of the full path model was then tested by combining all the probabilities using Fisher’s C statistic:

$$C=-2\sum_{i=1}^{k}\left(ln(p_{i}\right))$$

and comparing the resulting C value to a chi-square distribution with 2k degrees of freedom, where k = the total number of independencies and p_i_ = the null probability associated with independence claim i (S4 Table [79]). After determining that the correlational structure of the path model fit the data, we obtained individual path coefficients for the hypothesized paths by fitting a series of linear mixed effect models for each response variable in the revised path model (Fig 3A). We adopted the model selection approach developed by Shipley [81], using the Akaike Information Criterion (AIC) formulated for d-sep tests to evaluate the relative statistical support for all subsets of the full path model (Fig 3A). This produced a set of 3,255 candidate path models (available in the data repository). We considered all paths included in the most supported candidate path model (with the lowest AIC) to have strong support and we considered other paths included in models within 2 AIC of the most supported model (ΔAIC ≤ 2) to have moderate support. Paths that were only included in models with ΔAIC > 2 were considered to be unsupported. Path analysis was conducted in R version 4.2.2 [82], using the *piecewiseSEM* package (version 2.1.2; [83, 84]) and *nlme* package (version 3.1.160; [85, 86]).

Prior to model selection, we assessed the fit of all global models by examining residual plots. Residuals did not appear to violate assumptions of a Gaussian error distribution or homogeneity. We assessed the potential influence of multicollinearity by examining the Pearson correlation coefficients between all pairs of continuous predictors (S2 Fig) and, also pairwise relationships among predictor variables using linear mixed models (see the data repository). Pairwise correlations between predictor variables in the path model were all below the typically-accepted threshold of |r| = 0.7 [87].

#### Effects of land management type on gamma diversity

Because the abundance of calanoids (6 were keyed to species, 2 were unknown beyond Calanoida, 5% of all species, 20.7% of all individuals) was estimated using different methods, and the timing of sampling and methods for anostracans (3 species, 1.9% of all species, 2.5% of all individuals) were different than for the other taxa, we converted the counts for all taxa to a binary incidence response (presence/absence). We estimated aquatic invertebrate species richness in each land management type (gamma diversity) using sample incidence-based data with the R package iNEXT [88, 89], version 3.0.0, standardizing based on a sample size of 10; this implied rarefying species richness estimates for minimum tillage and organic sites (n = 11, each respectively) and extrapolating them for conventional and perennial cover sites (n = 9 each respectively) [90, 91]. We used non-overlapping 95% confidence intervals for diversity curves and point estimates as a conservative criterion of statistical difference [92].

#### Effects of land management type, landscape structure, and water quality on beta diversity and community composition (direct gradient analysis)

For these analyses, we disentangled the contributions of species turnover (i.e., spatial species replacement) and nestedness (i.e., richness differences) from the total compositional dissimilarity with R package betapart [93, 94], version 1.5.6 [95] using the incidence-based data (see previous section). We found that turnover alone accounted for ~79% of the total dissimilarity in aquatic invertebrate communities, whereas nestedness accounted for only ~21%. Therefore, all of our subsequent analyses were based on within-management type turnover-only dissimilarity (Simpson’s dissimilarity) of total compositional dissimilarity (Sörensen distance) and we used this to assess within- and between-management type variation in beta diversity. We assessed within-management type turnover using an analysis of homogeneity of multivariate group dispersion or variance [96] with the Tukey HSD correction to minimize family-wise type I error rate.

We also assessed land management type effects on species composition, controlling for nutrients, turbidity, cropland cover and wetland cover with distance-based redundancy analyses (db-RDA) in the R package *vegan* [97, 98], version 2. 6–4 [98]. To account for the spatial clustering of sites (space) and potential positive spatial autocorrelation within this analysis, we used distance-based Moran’s eigenvector maps (db-MEMS) [99–101] estimated using the *adespatial* R package, Version 0.3–2 [102]. We first calculated Euclidean distances based on the latitude and longitude coordinates of the ponds. We then estimated db-MEMs using the Euclidean method for positive autocorrelation, then sub-selecting db-MEMs based on forward selection; this resulted in four db-MEMs which we included in the db-RDA and variance partitioning (below). For the db-RDA, we performed permutation tests with 9,999 iterations to assess overall ordination significance, and the marginal effects of constraining terms (i.e., the effect of an individual term when all other terms are included in the model). To further investigate the role of land management type in relation to other factors, including spatial variation, we used variance partitioning [103, 104]. This enabled us to separate out the collective and individual effects of land management type, local factors (nutrients, turbidity), landscape factors (cropland, wetland), and space (db-MEMs) on aquatic invertebrate communities. Note that for comparative purposes we also ran db-RDA and variance partitioning models without space and include these in the Supplementary material (S3 and S4 Figs., S8 Table).

#### Indicator species

To identify the strength of species associations with land management types, we calculated the point bi-serial correlation coefficient (rho) and indicator species index (IndVal) with R package *indicspecies* [60], version 1.7.12 [105] and evaluated statistical significance with 9,999 Monte Carlo permutations.

#### *Post hoc* analyses

To investigate spatial patterns of covariates in relation to land management type, we ran a principal component analysis (PCA) on the environmental variables, and created plots of biophysical variables (conductivity and wetland area, early and late season water depth). We also plotted cropland area against open water separately for sites within each land management type to determine if there were any differences in the crop area–open water relationships among management types, and to enable us to speculate on causative mechanisms that could help to explain our results. To further investigate taxonomic differences among land management types, we performed a canonical analysis of principal coordinates (CAP [106]) using the software Permanova + for Primer [107], to maximize differences among land management types in ordination space and assess the predictive ability in assigning sites to specific land management groups [80]. Results of these *post hoc* analyses are provided in the Supplementary material (S5–S7 Figs).

## Results

A total of 159 taxa and 34,889 individuals was counted (73 were identified to species and 75 to genus or family), with 105, 95, 90 and 105 taxa on perennial cover, organic, minimum tillage, and conventional land management types, respectively (S1 Table).

### Direct and indirect effects of farm management type, landscape structure, and water quality on invertebrates

The independence test indicated that we should add paths between land management type and cropland, and between turbidity and nutrients. Note that the linkage between turbidity and nutrients was not an *a priori* prediction and could have been added in either direction (thus, this should not be interpreted as there being an indirect effect of turbidity on invertebrates via nutrients). After adding these linkages, the Fisher’s C statistic demonstrated that the correlational structure of the path model did not significantly differ from that of the data (S4 Table).

We found support for several of our predicted relationships (Fig 3A; S5 Table). Cropland cover had a positive effect on nutrient levels and turbidity. There were negative associations between both turbidity and nutrient levels and aquatic invertebrate richness, with greater support for nutrients. We also detected indirect relationships between land management type and invertebrate richness via an association between land management type and cropland, and associations between land management type and nutrient levels and turbidity. There was also a positive, direct effect of cropland area on richness. Note that here we refer to a direct effect as the effect from one predictor directly to aquatic invertebrate richness and an indirect effect as the effect of one predictor on richness via an intermediate predictor. Overall, the most strongly supported path from land management type to the response variable was via cropland → nutrients → aquatic invertebrate richness (S5 Table; Fig 3A and 3B). Moderate support (within 2 AICs of the most supported model) was also found for the land management type → cropland → turbidity → aquatic invertebrate richness path, the land management type → turbidity → aquatic invertebrate richness path and land management type → nutrients → aquatic invertebrate richness path (Fig 3A). Finally, there was moderate support for a direct path from cropland cover → aquatic invertebrate richness. Summary statistics from the most supported path model, i.e., the model with the smallest AIC are shown in S6 Table.

### Effects of land management type on gamma diversity

**Fig 4:**
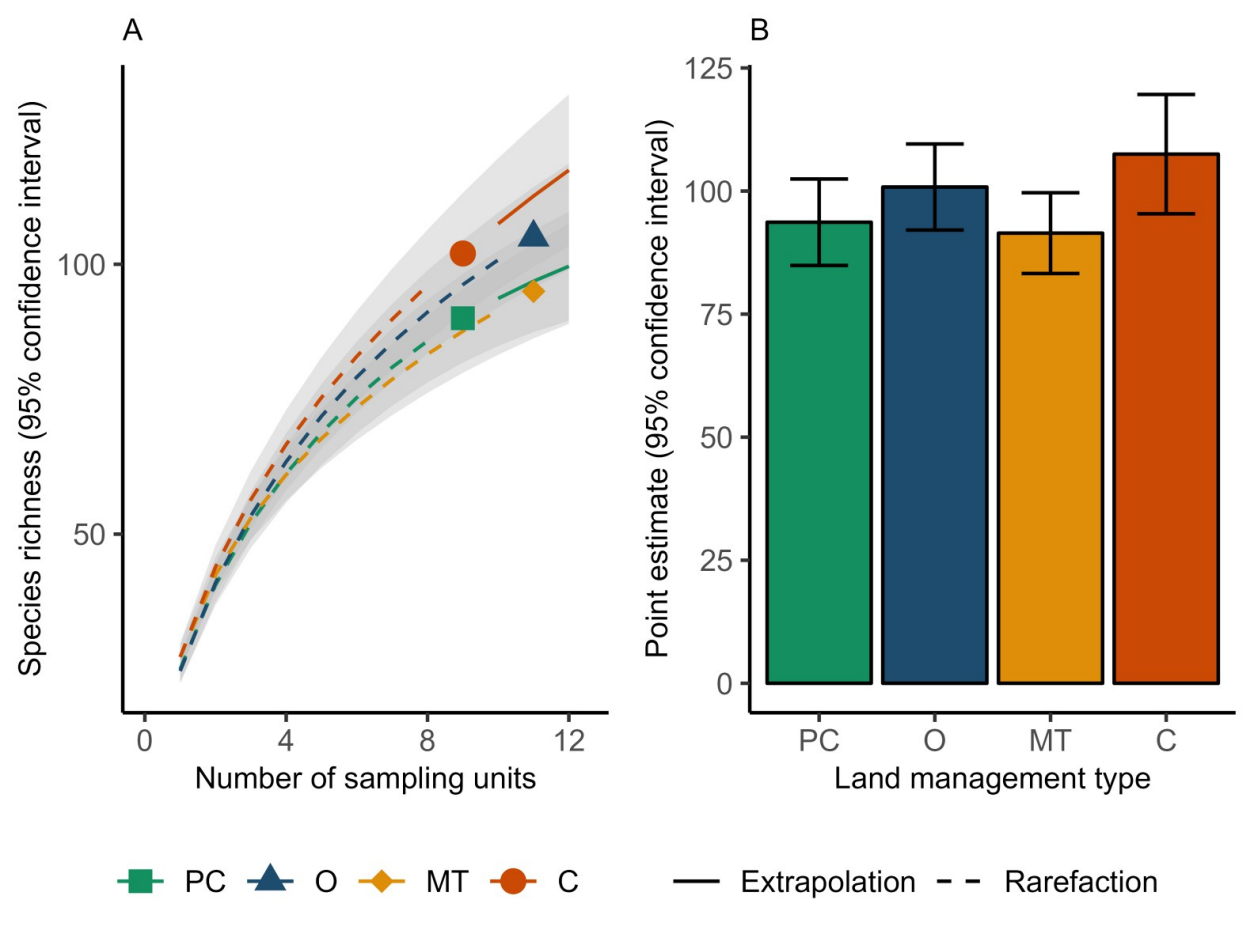
Gamma diversity of aquatic invertebrates in wetlands on four land management types in Saskatchewan (PC = perennial cover; O = organic; MT = minimum tillage; C = conventional), corrected for sampling coverage. a) HN q = 0 (richness) diversity curve per land management type with 95% confidence intervals and b) Point estimates for 10 sampling units per land management type with 95% confidence intervals. Point estimates were extrapolated for PC and C, and rarefied for O and MT.

We found no significant difference among land management types for estimated richness after correcting for the number of samples from each land management type (Fig 4A and 4B).

### Effects of land management type, water quality, and landscape context on beta diversity and community composition

**Fig 5:**
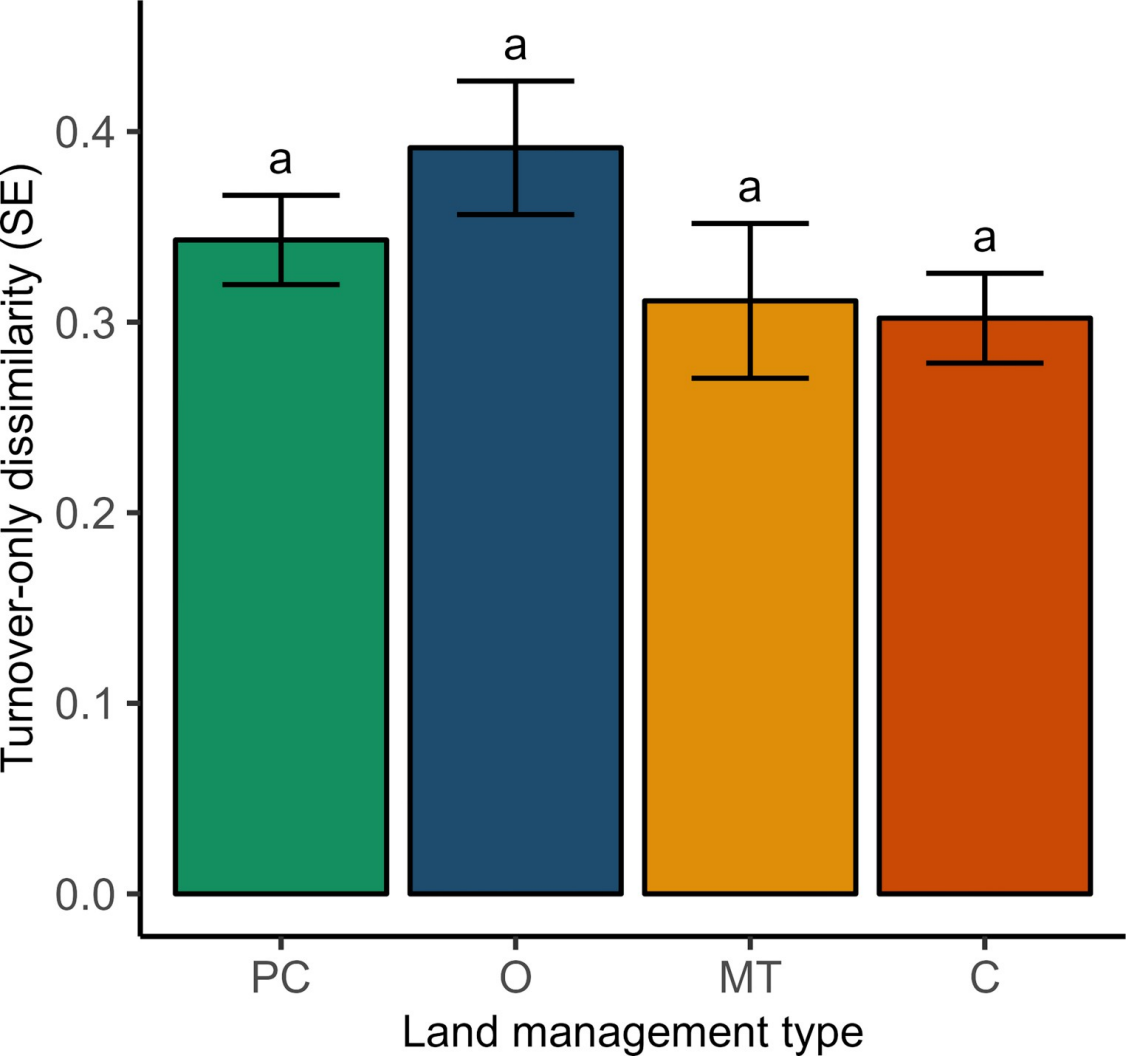
Effects of land management type on within-management-type beta diversity for presence/absence of aquatic invertebrates in Saskatchewan wetlands, using the turnover only component of total dissimilarity (Simpson dissimilarity). Bars show means ± SE. The population means of land management types were not significantly different at alpha = 0.05 (Tukey HSD method). Land management types: PC = perennial cover; O = organic; MT = minimum tillage; C = conventional.

We detected no significant differences in within-management β-diversity of aquatic invertebrates among land management types (F_3,36_ = 1.553, P = 0.2176; Fig 5). However, in terms of absolute values the largest distance from the centroid (highest β-diversity) was for organic land management sites (mean distance to centroid = 0.391) followed by perennial cover (0.343), minimum tillage (0.311), and conventional sites (0.302).

**Fig 6:**
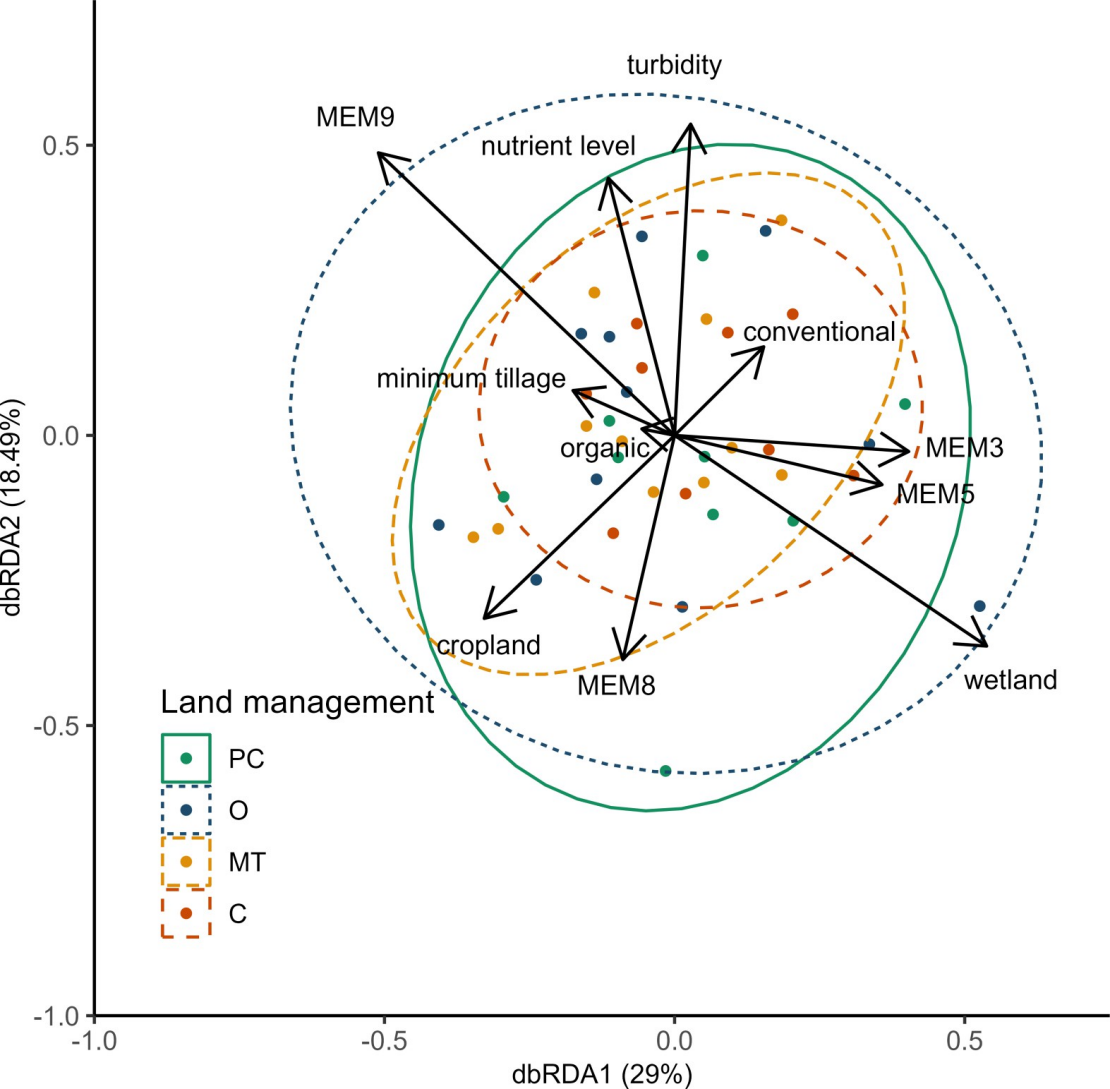
Distance-based redundancy analysis (db-RDA) showing the relationships between turnover-only dissimilarity of aquatic invertebrate species and land management type, water quality (total nutrient levels and turbidity), and landscape structure (cropland and wetland cover). Ellipses show 95% contours for land management types, with a clear trend in ellipse size (β diversity; conventional sites had the smallest ellipse, followed by minimum tillage, perennial cover and organic sites). The vectors show the direction and strength of relationship (length) in the ordination. Land management types: PC = perennial cover; O = organic; MT = minimum tillage; C = conventional. MEM3, 5, 8, and 9 = distance-based Moran’s eigenvector maps.

Land management type, cropland cover and wetland cover all had significant effects on turnover-only dissimilarity of aquatic invertebrate species in the db-RDA model (Fig 6). Three of the four db-MEMs were also significant in this analysis (MEM5, MEM3, MEM9; Table 1). We note also that including space (db-MEMs) in the db-RDA model increased the variance explained (adjusted r^2^) from 9% to 20%, indicating that spatial distance and unexplained factors at the landscape scale contributed a substantial component of the variation in aquatic invertebrate communities (S3 Fig, S8 Table).

**Table 1:** Distance-based redundancy analysis testing for differences in aquatic invertebrate composition (turnover-only dissimilarity) among land management types and effects of landscape, water quality and distance-based Moran’s eigenvector maps (MEMs) variables at 40 wetland sites in Saskatchewan. Significance ** P < 0.01; * P < 0.05.

Source	Df	SS	F	P
Cropland	1	0.274	2.712	0.0062 **
Wetland	1	0.255	2.518	0.0100 **
Nutrient levels	1	0.089	0.880	0.5682
Turbidity	1	0.137	1.353	0.2438
Land management type	3	0.565	1.864	0.0107
MEM5	1	0.201	1.984	0.0499 *
MEM3	1	0.288	2.848	0.0041**
MEM8	1	0.096	0.945	0.5240
MEM9	1	0.237	2.345	0.0167 *
Residuals	28	2.831		

**Fig 7:**
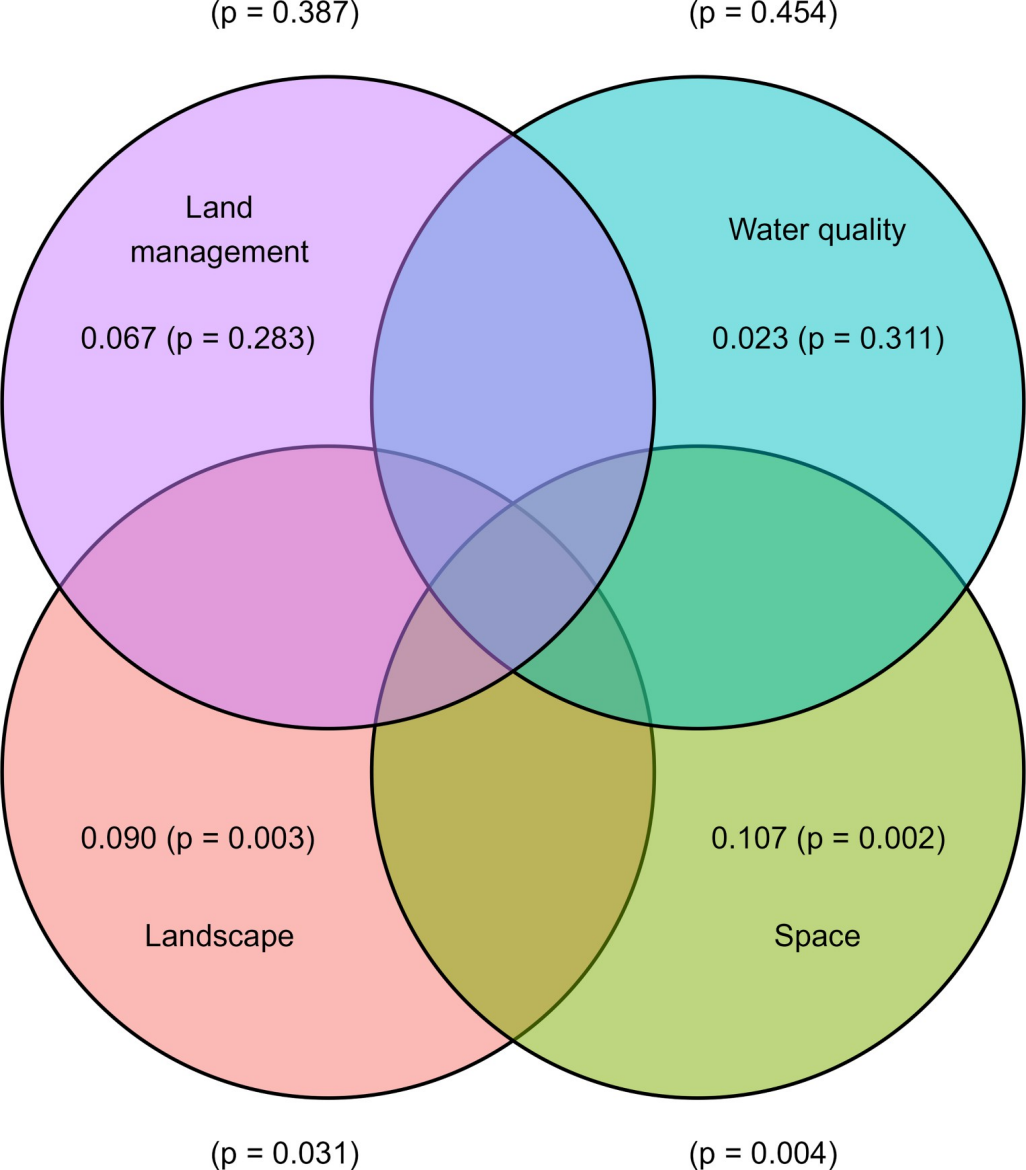
Variance partitioning of aquatic invertebrate beta diversity on different land management types in Saskatchewan. Diagram shows beta-diversity explained by land management type, landscape, water quality and space covariates across 40 sites. Values within ellipses are the conditional effects after controlling for other covariates (% variation explained and associated p-value). P-values outside ellipses for each covariate are the significances of the marginal effects. Only cases where the % variation was > 0.005 are displayed. The indicator species analysis (r.g.) identified the following taxa as being associated with specific land management types: perennial cover (*Tanytarsus sp*., [Diptera: Chironominae] test statistic = 0.426, P = 0.0497); minimum tillage (Enchytraeidae [Annelida: Oligochaeta], test statistic = 0.437, P = 0.0421); and conventional (*Cymatia americana* [Hemiptera: Corixidae] test statistic = 0.420, P = 0.0904, ns). No indicators were identified for organic farms alone. Several indicator species were identified that characterized more than one land management type. For example, for perennial cover and minimum tillage sites, *Limnephilus* sp. (Trichoptera: Limnephilidae) was the indicator (test statistic = 0.431, P = 0.0528). In the case of organic, minimum tillage and conventional farms, *Lestes unguiculatus* (Odonata: Lestidae [test statistic 0.397, P = 0.06624]) was an indicator. Interestingly, *Lestes* spp. and other Odonata are often used as indicators of wetland condition [108, 109], and possibly surrogates for overall invertebrate diversity (K. McLean, pers. comm.). Finally, for organic, minimum tillage and conventional farms, *Rhantus* sp. (Coleoptera: Dytiscidae) was an indicator (test statistic = 0.398, P = 0.0726).

Variance partitioning also demonstrated that space (db-MEM) alone explained a significant amount of the variation in invertebrate communities (Fig 7). Landscape level factors (i.e., wetland and cropland) also explained a significant amount of the variation in invertebrate communities. See S4 Fig. for variance partitioning results for the comparative db-RDA without space.

## Discussion

### Crop cover, nutrients and turbidity mediated the effects of land management type on aquatic invertebrate richness

Our structural equation model revealed indirect pathways for the effect of agricultural land management type on aquatic invertebrate richness, with effects via the cropland (+) → nutrients (–) and turbidity (–) → aquatic invertebrate richness paths (Fig 3A). These indirect effects of land management type on invertebrates are consistent with our general expectation that conventional farming has more negative effects on aquatic invertebrates than organic and minimum tillage farming, with the best outcomes for aquatic invertebrates when land was managed for perennial cover. This is because cropland cover was positively associated with nutrient levels and turbidity, which in turn had strong, negative effects on richness, and the most and least positive associations between land management type and cropland cover were for conventional and perennial cover, respectively. This was presumably due to the greater runoff of sediment and fertilizers in landscapes with more land in crop production. Interestingly, our path analysis revealed an effect of land management on aquatic invertebrate richness that would otherwise have been missed; had we only looked at the simple direct relationship between land management type and aquatic invertebrate richness (S7 Table), we would have found no statistical support for our predictions.

One could suggest that the lack of a direct effect of land management type on aquatic invertebrates may reflect the fact that wetlands embedded within landscapes using different land management types can actually have similar pesticide levels, despite differences in the amounts/types of pesticides applied. For example, Donald et al. (2018) found that levels of five herbicides (MCPA, bromoxynil, 2,4-D, dichlorprop, and dicamba) did not differ significantly between organic and minimum tillage farms on a subset of wetlands included in this study. Ideally, we would have evaluated the role of herbicides in mediating the response of invertebrates to land management types. However, information on herbicide use was only available for a subset of the wetlands (n = 29; [110]), and we therefore decided not to reduce our sample size further by including pesticides as a covariate in statistical models.

#### Nutrients more strongly mediated the effects of land management type and landscape context on aquatic invertebrate richness than turbidity

We found strong support for the direct effect of nutrients on aquatic invertebrate richness and nutrients mediated effects of land management type and cropland on richness (Fig 3). A recent meta-analysis demonstrated that N and P had consistently negative impacts on invertebrate abundance but in the case of species richness effects were weaker or inconclusive [45], perhaps partly due to the paucity of case studies for this community response. Another explanation, as speculated by [45],is that most studies have used total species richness as a measure of diversity rather than turnover (beta diversity); species negatively affected by N and P enrichment could be replaced by those that flourish under such conditions leading to no net changes in species richness. Many studies in the literature demonstrate toxic effects of N and P on aquatic organisms (e.g., [111]).

In contrast, evidence for the direct effect of turbidity on richness was weaker and, although moderate support was found for the indirect path from cropland cover to richness via turbidity, this relationship was much weaker than the relationship with cropland cover via nutrient level (Fig 3, S5 Table). Previous studies have found that turbidity can have mixed effects on aquatic invertebrates (sometimes positive, sometimes negative; [59, 112]).

We found moderate support for the expected relationships between land management type and both nutrient levels and turbidity. We observed that total nutrient level was highest on minimum tillage sites and lowest on perennial cover sites. While minimum tillage is considered an agricultural conservation practice to reduce soil erosion, it has also been associated with increased losses of phosphorus ([32, 113], We observed that turbidity was highest in wetlands on perennial cover sites where levels were almost twice the levels found at minimum tillage sites (S2 Table). In contrast, a previous study comparing sedimentation and turbidity under different farm management practices found that more intensive farming increased sedimentation [16]. In our case, possible explanations include summer haying followed by fall cattle grazing which were typical practices on some perennial cover sites. Cattle grazing can have a detrimental effect on aquatic invertebrates through manure [114] and causes disturbance through trampling as well as reduced emergent vegetation and increased runoff resulting in higher sedimentation levels and subsequent turbidity [114, 115].

### Unexpected effects of cropland on aquatic invertebrate richness

Landscape cropland cover had a direct positive effect on aquatic invertebrate richness, although we note that this path had only moderate support. The lack of strong support for an effect of cropland cover on richness is consistent with previous studies. While the densities of some pollution-tolerant groups of aquatic invertebrates (e.g., Chironomidae) may increase with expansion of surrounding crop area [116], generally studies suggest that cropland amount may not necessarily be a significant factor influencing aquatic invertebrate community structure or densities, e.g., [117]. In some cases, the lack of an effect of cultivated land around wetlands may possibly be due to methodological issues; for example, Gleason and Rooney [117] pooled agricultural disturbance from cultivation for cropping, with pasture for grazing, each of which may have very different impacts on aquatic invertebrates.

In our study, the positive effect of increased cropland cover could be due to several different mechanisms. First, the amalgamation of wetlands in cropped areas through enhanced natural (runoff) and artificial drainage (filling/levelling) creates larger and deeper water bodies [118], which in turn increases pond permanence, a key driver for aquatic invertebrate communities in the PPR [76]. For example, pond permanence influences the degree to which wetlands can be used for specific life history stages for aquatic invertebrates, such as larval forms. In addition, species which do not possess dessication-resistant traits may be better able to survive in such water bodies [27, 76]. Second, the spatial extent, depth and hydroperiod of wetlands has a pivotal influence on aquatic invertebrates [76], and so these larger wetlands can have a positive effect on aquatic invertebrate richness and abundance. In some areas, such as in North Dakota, larger wetlands tended to have higher alpha diversity [27], and increased surface water connectivity via open drainage can increase dispersal pathways [116, 119]. However, if increased wetland size was the mechanism underlying the positive effects of cropland in our study, we would have expected to see a path supported between cropland and wetland, and for wetland area to have a positive and not (albeit unsupported) negative effect on aquatic invertebrate richness.

### Less support for effects of land management type on gamma and beta diversity

Unlike the path analysis—where we detected strong support for the (indirect) effect of land management type on aquatic invertebrates—there was little or no statistical support for the effect of management type on gamma diversity or simple tests of beta diversity (the test for homogeneity of multivariate dispersions). Yet land management type had a significant effect on turnover-only dissimilarity in the db-RDA, whether or not we controlled for space. Furthermore, we found that landscape level factors and spatial location explained more of the compositional variation in aquatic invertebrates than local factors (i.e., water quality within the wetland). This contrasts with some previous findings, where local factors (e.g., fish communities or macrophytes) have generally been found to be more important to aquatic invertebrate communities than landscape-level factors [49, 116, 120, 121].

The lack of differences in gamma and beta diversity between land management types in our study may be due to a combination of other unmeasured variables, including intrinsic and extrinsic factors (see S1 Appendix). For example, it is possible that there was a diluted effect of land management type on gamma and beta diversity of aquatic invertebrates because pesticides in wetlands in the prairies are widely dispersed [110, 122, 123]. While synthetic pesticides were not used at perennial cover and organic sites, many herbicide compounds are highly soluble and vaporize in the atmosphere at the regional scale. Even if aquatic invertebrates were sensitive to practices on different land management types (and pesticide use in particular), the ubiquitous distribution of pesticides across land management types could lead to some homogenization of communities. More generally, wetlands have historically been subjected to many disturbances (e.g., sedimentation, changes in nutrient balance) to which they show resilience, and upland cultivation may mimic those effects. If this is the case, it could have led to little detectable change in communities among landscapes with different land use intensities [116].

### Management implications

We recognize that agricultural practices have changed considerably since 1996 when our study was conducted. Without repeating a contemporary study of the four different land management types, it is impossible to state with certainty that our conclusions would be the same. Nevertheless, understanding the effects of these land management types in the 1990s is still important today, given that current aquatic macroinvertebrate community diversity and composition would be to some degree dependent on historical management practices. It also has added value by providing an historical reference that could be used to compare with more contemporary surveys. Such comparisons may help us to better understand the impacts of changing agricultural practices on aquatic systems embedded in agricultural landscapes.

Our results suggest two possible strategies for effective conservation action for aquatic invertebrates in prairie wetlands. First, reducing nutrient inputs appears to be beneficial, as well as reducing cropland erosion to decrease sedimentation. Second, our findings imply a benefit of perennial cover farming for aquatic invertebrate richness relative to the other three land management types considered in this study. However, we caution that the direct effect of land management type was not a supported path (ΔAIC > 3.2) and the only reason we detected a land management type effect was because we modeled its indirect effects on richness via cropland cover, nutrients, and turbidity. Further analyses of functional traits may elucidate some of the patterns we uncovered and provide additional insight on responses of invertebrate communities to environmental factors at our study sites.

## Supporting information

S1 Table Complete list of taxa found in 40 wetlands on four land management types on farms in Saskatchewan. (DOCX)

S2 Table Full list of water quality variables measured in 40 wetlands, with mean ± SE values for sites in each land management type. Unexpectedly, levels of nitrogen were highest on organic farms and lowest on conventional sites. However, most forms of phosphorus were highest on minimum tillage farms (except for particulate phosphorus which was highest on organic farms).
(DOCX)

S3 Table Amounts of different land cover types found within a 1 km buffer around wetlands, with mean ± SE (units = ha) for sites in each land management type. Although cropland cover was highest on conventional farms (and about twice that on perennial cover sites), these farms also had the highest average cover of tall shrubs (which intercept runoff from snowmelt).
(DOCX)

S4 Table All conditionally independent pairs of variables, structured as independence claims, implied by the path model of the predicted relationships among land management type, water quality measures, landscape structure, and aquatic invertebrate richness (Fig 3A), and the associated models constructed to test the independence claims. Also shown are the null probabilities (*p* values) used to calculate Fisher’s *C* statistic to test the correlational structure of the full path model in Fig 1. The independence claim notation identifies the tested pair of variables in parentheses, followed by the variables that were statistically controlled for while testing the independence between the pair, in curled brackets. Two arrows were added to the path diagram (between crop and management and nutrients and turbidity) following the independence test based on this set of claims. With these arrows added, the p-value of the Fisher’s C statistic for the path model was 0.74.
(DOCX)

S5 Table Results of model selection, comparing across all subsets of the full path model (see Fig 3A). Relationships were modeled by linear mixed models with cluster as a random factor. Presented are the Akaike Information Criterion (AIC) and change in AIC (relative to the most supported model; ΔAIC) for all models with ΔAIC ≤ 2.
(DOCX)

S6 Table Summary statistics from the most supported path model, i.e., the model with the smallest Akaike Information Criterion (see S5 Table, above, for details). The p-value of the Fisher’s C statistic for the path model was 0.961.
(DOCX)

S7 Table Results of modeling aquatic invertebrate species richness as a function of land management type, using a generalized linear model with cluster as a random effect. (DOCX)

S8 Table Distance-based redundancy analysis testing for differences in aquatic invertebrate composition (turnover-only dissimilarity) among land management types and effects of landscape and water quality variables at 40 wetland sites in Saskatchewan (not including space). Significance ** P < 0.01; * P < 0.05.
(DOCX)

S1 Fig Boxplots showing biophysical characteristics of wetlands in different land management types, including total wetland area within 65 ha, conductivity, water depth in May (early season) and water depth in July (late season). PC = perennial cover; O = organic; MT = minimum tillage; C = conventional. Box and whisker plots summarizing median, inter-quantile range, 1.5 times the inter-quartile range, and extreme values. The population means of land management types with the same letter are not significantly different at alpha = 0.05 (Tukey HSD method).
(TIFF)

S2 Fig Matrix scatterplot showing pairwise Pearson correlations between continuous predictor variables (total nutrient levels, turbidity, cropland cover, and wetland cover). (TIFF)

S3 Fig Distance-based redundancy analysis (db-RDA) showing the relationships between turnover-only dissimilarity of aquatic invertebrate species and land management type, water quality (total nutrient levels and turbidity), and landscape structure (cropland and wetland cover)–not including space. Ellipses show 95% contours for land management types. The vectors show the direction and strength of relationship (length) in the ordination. Land management types: PC = perennial cover; O = organic; MT = minimum tillage; C = conventional.
(TIFF)

S4 Fig Variance partitioning of aquatic invertebrate beta diversity on different land management types in Saskatchewan. Diagram shows beta-diversity explained by land management type, landscape and local covariates across 40 sites (not including space). Values within ellipses are the conditional effects after controlling for other covariates (% variation explained and associated p-value). P-values outside ellipses for each covariate are the significances of the marginal effects.
(TIFF)

S5 Fig Principal Component Analysis (PCA) of covariates in relation to land management type, with 95% confidence ellipses for the four land management types. Salinity was derived from Surficial geology of Saskatchewan soil maps (Government of Canada 2023: Agri-Environmental Indicator–Risk of Soil Salinization. https://open.canada.ca/data/en/dataset/8e2583c8-af71-4873-8cca-8081db789121 and https://agriculture.canada.ca/en/agricultural-production/soil-and-land/soil-salinization-indicator). Land management: PC = perennial cover; O = organic; MT = minimum tillage; C = conventional). Note that salinity was correlated with cropland cover but salinity levels did not approach toxicity levels for invertebrates (K. McLean, USGS, pers. comm.).
(TIFF)

S6 Fig Relationship between wetland area (open water in 65 ha quarter sections that include the sampled wetland) and cropland cover for sites with different land management types (PC = perennial cover; O = organic; MT = minimum tillage; C = conventional). (TIFF)

S7 Fig Canonical analysis of principal coordinates (CAP) of presence/absence of aquatic invertebrate species (turnover-only dissimilarity) in wetlands by land management type (PC = perennial cover; O = organic; MT = minimum tillage; C = conventional). Some separation of land management types occurred on axes 1 and 2, with organic sites on the right side of the ordination and conventional sites on the left, but there was no significant difference among farm types (P > 0.1). The CAP analysis indicated that there was a high misclassification error in assigning wetlands to farm management types (30% correct, 70% misclassified). Minimum tillage had the highest percentage of correct classifications (45.5%). Many species appeared to be associated with these sites (e.g., *Notonecta undulata*, *Aeshna subarctica*, *Colymbetes sculptilis*; Pearson correlation 0. 3). See S1 Table for species abbreviations.
(TIFF)

S1 Appendix Additional discussion. (DOCX)
