# Supplementary material for: Crop cover and nutrient levels mediate the effects of land management type on aquatic invertebrate richness in prairie potholes
Source: PLoS One. 2024 Apr 16;19(4):e0295001. doi: 10.1371/journal.pone.0295001 (PMC11020495; doi:10.1371/journal.pone.0295001)
Supplement: S1 Table — (DOCX) [file pone.0295001.s001.docx]

| Phylum (subphylum) | Class (Subclass) | Order (Infraorder) | | | Family (Subfamily) | Species | Common name | Acronym | PC | O | MT | C |
| --- | --- | --- | --- | --- | --- | --- | --- | --- | --- | --- | --- | --- |
| Annelida | Clitellata (Hirudinea) | Arhynchobdellida | | | Erpobdellidae | *Erpobdella (Nephelopsis) obscura* | a leech | Nephelob | 0 | 0 | 0 | 1 |
| Annelida | Clitellata (Hirudinea) | Rhynchobdellida | | | Glossophoniidae | *Glossiphonia complanata* | a leech | Glossicom | 0 | 0 | 0 | 1 |
| Annelida | Clitellata (Hirudinea) | Rhynchobdellida | | | Glossophoniidae | *Marvinmeyeria lucida* | a leech | Marvinlu | 1 | 1 | 1 | 1 |
| Annelida | Clitellata (Oligochaeta) | Lumbriculida | | | Lumbriculidae | *Lumbriculus* sp. | an aquatic worm | Lumbri_s | 0 | 1 | 0 | 0 |
| Annelida | Clitellata (Oligochaeta) | Enchytraeidae | | | Enchytraeidae | Enchytraeidae sp. | an aquatic worm (microdile oligochaete) | Enchyt_F | 1 | 0 | 1 | 0 |
| Annelida | Clitellata (Oligochaeta) | Naididae (Tubicinae) | | | Tubifidae | *Chaetogaster limnaei* | a Naidid worm | Chaetoli | 1 | 1 | 1 | 1 |
| Annelida | Clitellata (Oligochaeta) | Naididae (Tubicinae) | | | Tubifidae | *Nais* sp. | a naidid worm | Nais_sp | 1 | 1 | 1 | 1 |
| Annelida | Clitellata (Oligochaeta) | Naididae (Tubicinae) | | | Tubifidae | Tubificine worm | a naidid worm | Tubif_F | 1 | 1 | 1 | 1 |
| Arthropoda | Arachnida | Araneae | | | Araneidae | *Araneus sp. or Araneidae sp.?* | orbweaver spider | Aranea_O | 1 | 1 | 0 | 1 |
| Arthropoda | Arachnida | Sarcoptiformes | | | Hydrozetidae | *Hydrozetes* sp. | an oribatid mite | Hydroz_s | 0 | 1 | 1 | 0 |
| Arthropoda | Arachnida | Trombidiformes | | | Arrenuridae | *Arrenurus* sp. | a water mite | Arrenu_s | 0 | 0 | 1 | 1 |
| Arthropoda | Arachnida | Trombidiformes | | | Eylaidae | *Eylais* sp. | a water mite | Eylais_s | 1 | 1 | 1 | 1 |
| Arthropoda | Arachnida | Trombidiformes | | | Eylaidae or Hydrachnidae | *Hydrachna* sp. | a water mite | Hydrach_s | 1 | 1 | 1 | 1 |
| Arthropoda | Arachnida | Trombidiformes | | | Limnesiidae | *Limnesia* sp. | a water mite | Limnes_s | 1 | 1 | 0 | 1 |
| Arthropoda | Arachnida | Trombidiformes | | | Pionidae | *Piona* sp. | a water mite | Piona_s | 1 | 1 | 1 | 1 |
| Arthropoda | Arachnida (Acari) | Sarcoptiformes | | | Oribatida fam. | Oribatei sp. | an oribatid mite | Oribat_o | 1 | 0 | 0 | 0 |
| Arthropoda | Branchiopoda | Anostraca | | | Chirocephalidae | *Eubranchipus (Chirocephalopsis) bundyi* | Knobbedlip Fairy Shrimp | Chirocbu | 1 | 1 | 1 | 1 |
| Arthropoda | Branchiopoda | Anostraca | | | Chirocephalidae | *Eubranchypus intricatus* | Smoothlip Fairy Shrimp | Eubraninc | 1 | 1 | 1 | 1 |
| Arthropoda | Branchiopoda | Anostraca | | | Chirocephalidae | *Eubranchypus ornatus* | Ornate Fairy Shrimp | Eubranorn | 1 | 1 | 1 | 1 |
| Arthropoda | Branchiopoda | Laevicaudata | | | Chydoridae | *Alona* sp. | a crustacean | Alona_s | 0 | 0 | 0 | 1 |
| Arthropoda | Branchiopoda | Laevicaudata | | | Chydoridae | Chydoridae sp. | a branchiopod | Chydor_F | 1 | 1 | 0 | 1 |
| Arthropoda | Branchiopoda | Laevicaudata | | | Daphniidae | *Ceriodaphnia* sp. | a water flea | Ceriod_s | 0 | 0 | 1 | 1 |
| Arthropoda | Branchiopoda | Laevicaudata | | | Daphniidae | *Daphnia* sp. | a water flea | Daphni_s | 1 | 1 | 1 | 1 |
| Arthropoda | Branchiopoda | Laevicaudata | | | Daphniidae | *Scapholeberis* sp. (*mucronata*?) | a water flea | Scapho_s | 0 | 1 | 0 | 0 |
| Arthropoda | Branchiopoda | Laevicaudata | | | Daphniidae | *Simocephalus* sp. | a water flea | Simoc_s | 1 | 1 | 1 | 1 |
| Arthropoda | Branchiopoda | Laevicaudata | | | Lyncaeidae | *Lynceus brachyurus* | Holarctic Clam Shrimp | Lynceusb | 1 | 1 | 1 | 1 |
| Arthropoda | Collembola | Entomobryomorpha | | | Isotomidae | *Isotomurus* sp | a springtail | Isotom_s | 1 | 1 | 0 | 0 |
| Arthropoda | Collembola | Poduromorpha | | | Poduridae | *Podura aquatica* | Water Springtail | Poduaqu | 1 | 0 | 0 | 0 |
| Arthropoda | Insecta | Coleoptera | | | Caribidae | Carabid sp. | Carabid beetle | Carabid_F | 0 | 1 | 0 | 0 |
| Arthropoda | Insecta | Coleoptera | | | Chrysomelidae | *Pyrrhalta* sp. | a leaf beetle | Pyrrha_s | 0 | 1 | 0 | 1 |
| Arthropoda | Insecta | Coleoptera | | | Curculionidae | Curculionidae sp. | a weevil | Curcul_F | 0 | 1 | 1 | 0 |
| Arthropoda | Insecta | Coleoptera | | | Dytiscidae | *Hygrotus (Leptolambus) marklini (Hygrotus canadensis)* | Marklin's Diving Beetle | Hygrocan | 1 | 1 | 1 | 0 |
| Arthropoda | Insecta | Coleoptera | | | Dytiscidae | *Hydaticus aruspex (modestus)* | Haruspex Predaceous Diving Beetle | Hydatmod | 0 | 1 | 1 | 0 |
| Arthropoda | Insecta | Coleoptera | | | Dytiscidae | *Hydroporus fuscipennis (criniticoxis)* | diving beetle | Hydropcr | 1 | 1 | 1 | 0 |
| Arthropoda | Insecta | Coleoptera | | | Dytiscidae | *Hydroporus striola* | Common Boreal Water Beetle | Hydropst | 0 | 1 | 0 | 0 |
| Arthropoda | Insecta | Coleoptera | | | Dytiscidae | *Acilius sp.* | predaceous diving beetle | Aciliu_s | 0 | 1 | 0 | 0 |
| Arthropoda | Insecta | Coleoptera | | | Dytiscidae | *Agabus sp.* | Agabus beetle | Agabus_s | 1 | 1 | 0 | 1 |
| Arthropoda | Insecta | Coleoptera | | | Dytiscidae | *Hygrotus*(*Leptolambus*) *impressopunctatus* | Big-dot Diving Beetle | Hygroimp | 0 | 1 | 0 | 0 |
| Arthropoda | Insecta | Coleoptera | | | Dytiscidae | *Hygrotus*(*Leptolambus*) *patruelis* | diving beetle | Hygropat | 0 | 1 | 1 | 1 |
| Arthropoda | Insecta | Coleoptera | | | Dytiscidae | *Hygrotus* sp. | diving beetle | Hygro_s | 1 | 1 | 0 | 1 |
| Arthropoda | Insecta | Coleoptera | | | Dytiscidae | *Colymbetes sculptilis* | Sculptured Predaceous Diving Beetle | Colymbsc | 0 | 0 | 1 | 0 |
| Arthropoda | Insecta | Coleoptera | | | Dytiscidae | *Dytiscus* sp. | a diving beetle | Dytisc_s | 1 | 1 | 1 | 1 |
| Arthropoda | Insecta | Coleoptera | | | Dytiscidae | *Graphoderus perplexus* | Complex Predaceous Diving Beetle | Graphope | 0 | 0 | 0 | 1 |
| Arthropoda | Insecta | Coleoptera | | | Dytiscidae | *Graphoderus* sp. | a predaceous diving beetle | Grapho_s | 1 | 1 | 1 | 1 |
| Arthropoda | Insecta | Coleoptera | | | Dytiscidae | *Rhantus sericans (frontalis)* | Satiny Swimming Beetle | Rhantfro | 1 | 0 | 1 | 1 |
| Arthropoda | Insecta | Coleoptera | | | Dytiscidae | *Rhantus* sp. | a swimming beetle | Rhant_s | 1 | 1 | 1 | 1 |
| Arthropoda | Insecta | Coleoptera | | | Dytiscidae (diving beetles) | *Hygrotus sayi* | Say's Diving Beetle | Hygrosay | 0 | 1 | 0 | 0 |
| Arthropoda | Insecta | Coleoptera | | | Dytiscidae (diving beetles) | *Laccophilus biguttatus* | Two-spotted Predaceous Diving Beetle | Laccobig | 1 | 1 | 1 | 1 |
| Arthropoda | Insecta | Coleoptera | | | Dytiscidae (diving beetles) | *Liodessus affinis* | Kindred Predaceous Diving Beetle | Liodesaf | 1 | 0 | 0 | 0 |
| Arthropoda | Insecta | Coleoptera | | | Gyrinidae | *Gyrinus* sp. | a whirligig beetle | Gyrin_s | 1 | 0 | 1 | 1 |
| Arthropoda | Insecta | Coleoptera | | | Hydrophilidae | *Enochrus hamiltoni* | Hamilton's Water Scavenger | Enochham | 0 | 1 | 1 | 1 |
| Arthropoda | Insecta | Coleoptera | | | Hydrophilidae | *Enochrus* sp. | a water scavenger beetle | Enoch_s | 0 | 1 | 0 | 0 |
| Arthropoda | Insecta | Coleoptera | | | Haliplidae | *Haliplus* sp. | Crawling water beetle | Halip_s | 1 | 1 | 1 | 1 |
| Arthropoda | Insecta | Coleoptera | | | Haliplidae | *Peltodytes* sp. | a crawling water beetle | Peltod_s | 0 | 0 | 0 | 1 |
| Arthropoda | Insecta | Coleoptera | | | Hydraenidae | *Limnebius* sp. | a minute moss beetle | Limneb_s | 0 | 1 | 0 | 0 |
| Arthropoda | Insecta | Coleoptera | | | Hydrophilidae | *Berosus oregonensis* | Oregon Water Scavenger Beetle | Berosore | 0 | 1 | 0 | 1 |
| Arthropoda | Insecta | Coleoptera | | | Hydrophilidae | *Cercyon* sp. | a scavenger beetle | Cercyo_s | 0 | 0 | 1 | 0 |
| Arthropoda | Insecta | Coleoptera | | | Helophoridae | *Helophorus linearoides* | Linear Water Scavenger Beetle | Helophli | 1 | 1 | 1 | 1 |
| Arthropoda | Insecta | Coleoptera | | | Helophoridae | *Helophorus oblongus* | Oblong Pool Scavenger | Helophob | 1 | 0 | 0 | 0 |
| Arthropoda | Insecta | Coleoptera | | | Helophoridae | *Helophorus orientalis* | Oriental Water Scavenger | Helophor | 1 | 1 | 1 | 1 |
| Arthropoda | Insecta | Coleoptera | | | Hydrophilidae | *Hydrobius fuscipes* | Swampy Water Scavenger | Hydrobfu | 0 | 1 | 0 | 1 |
| Arthropoda | Insecta | Coleoptera | | | Hydrophilidae | *Hydrochara obtusata* | Weedy Pond Scavenger | Hydroch | 0 | 0 | 0 | 1 |
| Arthropoda | Insecta | Coleoptera | | | Hydrochidae | *Hydrochus currani* | Curran's Water Scavenging Beetle | Hydroccu | 0 | 0 | 1 | 0 |
| Arthropoda | Insecta | Coleoptera | | | Hydrochidae | *Hydrochus granulatus* | Granular Water Scavenging Beetle | Hydrocgr | 0 | 0 | 1 | 0 |
| Arthropoda | Insecta | Diptera | | | Ceratopogonidae | *Dasyhelea* sp. | a biting midge | Dasyhe_s | 0 | 1 | 1 | 0 |
| Arthropoda | Insecta | Diptera | | | Chaoboridae | *Chaoborus americanus* | a phantom midge | Chaobam | 1 | 1 | 1 | 1 |
| Arthropoda | Insecta | Diptera | | | Chaoboridae | *Charoborus crystallinus* | a phantom midge | Chaobcry | 1 | 1 | 1 | 1 |
| Arthropoda | Insecta | Diptera | | | Chironomidae | Chironomid sp. | a midge | Chiron_F | 0 | 1 | 0 | 1 |
| Arthropoda | Insecta | Diptera | | | Chironomidae (Chironominae) | *Chironomus sp.* | a midge | Chiron_s | 1 | 1 | 1 | 1 |
| Arthropoda | Insecta | Diptera | | | Chironomidae (Orthocladiinae) | *Cricotopus sylvestris* gp. sp. | a cricotopus chironomid | Cricosy_s | 1 | 1 | 1 | 1 |
| Arthropoda | Insecta | Diptera | | | Chironomidae (Chironominae) | *Dicrotendipes* sp. | a midge | Dicro_s | 1 | 0 | 0 | 0 |
| Arthropoda | Insecta | Diptera | | | Chironomidae (Chironominae) | *Endochironomus* sp | a midge | Endoch_s | 1 | 1 | 1 | 1 |
| Arthropoda | Insecta | Diptera | | | Chironomidae (Chironominae) | *Glyptotendipes* sp. | a midge | Glypto_s | 1 | 1 | 1 | 1 |
| Arthropoda | Insecta | Diptera | | | Chironomidae (Orthocladiinae) | *Limnophyes* sp. | a midge | Limnop_s | 1 | 0 | 0 | 0 |
| Arthropoda | Insecta | Diptera | | | Chironomidae (Chironominae) | *Parachironomus* sp. | a midge | Parach_s | 0 | 1 | 0 | 1 |
| Arthropoda | Insecta | Diptera | | | Chironomidae (Chironominae) | *Paratanytarsus* sp. | a midge | Parata_s | 1 | 1 | 1 | 0 |
| Arthropoda | Insecta | Diptera | | | Chironomidae (Chironominae) | *Phaenopsectra* sp. | a midge | Phaeno_s | 0 | 0 | 1 | 0 |
| Arthropoda | Insecta | Diptera | | | Chironomidae (Chironominae) | *Polypedilum* sp. | a non-biting midge | Polype_s | 1 | 0 | 1 | 0 |
| Arthropoda | Insecta | Diptera | | | Chironomidae | *Procladius sp.* | a midge | Proclad_s | 0 | 0 | 0 | 1 |
|  |  |  |  |  | (Tanypodinae) |  |  |  |  |  |  |  |
| Arthropoda | Insecta | Diptera | | | Chironomidae (Orthocladiinae) | *Psectrocladius* sp. | a midge | Psectr_s | 1 | 1 | 1 | 1 |
| Arthropoda | Insecta | Diptera | | | Chironomidae (Tanypodinae) | *Psectrotanypus* sp. | a midge | Psecta_s | 0 | 1 | 1 | 0 |
| Arthropoda | Insecta | Diptera | | | Chironomidae (Chironominae) | *Tanytarsus* sp. | a midge | Tanyta_s | 1 | 0 | 1 | 0 |
| Arthropoda | Insecta | Diptera | | | Chironomidae (Orthocladiinae) | *Acricotopus* sp. | non-biting midge | Acrico_s | 1 | 1 | 1 | 1 |
| Arthropoda | Insecta | Diptera | | | Chironomidae (Orthocladiinae) | *Corynoneura* sp. | a midge | Coryno_s | 1 | 1 | 0 | 1 |
| Arthropoda | Insecta | Diptera | | | Chironomidae (Orthocladiinae) | *Cricotopus or Orthocladius* sp. | a cricotopus or orthocladius chironomid | Cricoto_s | 0 | 1 | 0 | 1 |
| Arthropoda | Insecta | Diptera | | | Chironomidae (Tanypodinae) | *Ablabesmyia* sp. | a midge | Ablabe_s | 1 | 1 | 1 | 1 |
| Arthropoda | Insecta | Diptera | | | Chironomidae (Tanypodinae) | *Derotanypus* sp. ? | non-biting midge | Derota_s | 0 | 0 | 1 | 0 |
| Arthropoda | Insecta | Diptera | | | Culicidae | *Aedes* sp. | a mosquito | Aedes_s | 0 | 1 | 0 | 1 |
| Arthropoda | Insecta | Diptera | | | Culicidae | *Anopheles earlei* | Earle's Mosquito | Anophear | 1 | 1 | 0 | 0 |
| Arthropoda | Insecta | Diptera | | | Culicidae | Culicidae sp. | a mosquito | Culic_F | 1 | 0 | 0 | 0 |
| Arthropoda | Insecta | Diptera | | | Ephydridae | Ephydridae sp. | a shore fly | Ephydr_F | 0 | 1 | 0 | 1 |
| Arthropoda | Insecta | Diptera | | | Sciomyzidae | *Sciomyza* sp. | a marsh fly | Sciomy_F | 0 | 0 | 1 | 0 |
| Arthropoda | Insecta | Diptera | | | Stratomyidae | *Hedriodiscus* sp. | a soldier fly | Hedrio_s | 0 | 1 | 0 | 0 |
| Arthropoda | Insecta | Diptera | | | Stratomyidae | *Odontomyia* sp. | a soldier fly | Odonto_s | 1 | 0 | 1 | 0 |
| Arthropoda | Insecta | Diptera | | | Tipulidae | *Prionocera* sp. | a true cranefly | Priono_s | 1 | 1 | 0 | 1 |
| Arthropoda | Insecta | Diptera (Brachycera) | | | Muscidae | *Muscomorpha* sp. | a house fly | Musco | 1 | 1 | 1 | 1 |
| Arthropoda | Insecta | Ephemeroptera | | | Baetidae | *Callibaetis sp.* | a mayfly | Callib_s | 0 | 0 | 0 | 1 |
| Arthropoda | Insecta | Ephemeroptera | | | Caenidae | *Caenis youngi* | Young's Small Square-gilled Mayfly | Caenisyo | 1 | 0 | 0 | 1 |
| Arthropoda | Insecta | Hemiptera | | | Corixidae | *Callicorixa audeni* | a water boatman | Callicau | 1 | 1 | 1 | 1 |
| Arthropoda | Insecta | Hemiptera | | | Corixidae | Corixid species | a water boatman | Corix_F | 1 | 1 | 1 | 1 |
| Arthropoda | Insecta | Hemiptera | | | Corixidae | *Cymatia americana* | a water boatman | Cymatam | 0 | 0 | 0 | 1 |
| Arthropoda | Insecta | Hemiptera | | | Corixidae | *Hesperocorixa atopodonta* | a water boatman | Hesperat | 0 | 0 | 1 | 0 |
| Arthropoda | Insecta | Hemiptera | | | Corixidae | *Hesperocorixa vulgaris* | a water boatman | Hespvulg | 0 | 0 | 0 | 1 |
| Arthropoda | Insecta | Hemiptera | | | Corixidae | *Sigara alternata* | a water boatman | Sigaralt | 0 | 0 | 0 | 1 |
| Arthropoda | Insecta | Hemiptera | | | Corixidae | *Sigara bicoloripennis* | a water boatman | Sigarbic | 0 | 1 | 0 | 0 |
| Arthropoda | Insecta | Hemiptera | | | Corixidae | *Sigara decoratella* | a water boatman | Sigardec | 0 | 1 | 0 | 1 |
| Arthropoda | Insecta | Hemiptera | | | Corixidae | *Trichocorixa verticalis interiores* | a water boatman | Trichovi | 0 | 0 | 1 | 0 |
| Arthropoda | Insecta | Hemiptera | | | Gerridae | *Gerris buenoi* | a water strider | Gerrisbu | 0 | 1 | 1 | 1 |
| Arthropoda | Insecta | Hemiptera | | | Gerridae | *Gerris pingreensis* | a water strider | Gerrispi | 1 | 0 | 0 | 1 |
| Arthropoda | Insecta | Hemiptera | | | Gerridae | *Gerris* sp. | a water strider | Gerris_s | 1 | 1 | 1 | 0 |
| Arthropoda | Insecta | Hemiptera | | | Notonectidae | *Notonecta borealis* | a backswimmer | Notonbor | 0 | 0 | 0 | 1 |
| Arthropoda | Insecta | Hemiptera | | | Notonectidae | *Notonecta* sp | a backswimmer | Notone_s | 1 | 1 | 1 | 1 |
| Arthropoda | Insecta | Hemiptera | | | Notonectidae | *Notonecta undulata* | a backswimmer | Notoneun | 0 | 0 | 1 | 0 |
| Arthropoda | Insecta | Hemiptera | | | Veliidae | *Microvelia buenoi* | a small water strider | Microvbu | 0 | 1 | 0 | 0 |
| Arthropoda | Insecta | Odonata | | | Aeshnidae | *Aeshna* sp. | Darner species | Aeshna_s | 1 | 0 | 0 | 1 |
| Arthropoda | Insecta | Odonata | | | Aeshnidae | *Aeshna subarctica* | Subarctic Darner | Aeshnasu | 0 | 0 | 1 | 0 |
| Arthropoda | Insecta | Odonata | | | Coenagrionidae | *Coenagrion resolutum* | Taiga Bluet | Coenares | 1 | 1 | 0 | 1 |
| Arthropoda | Insecta | Odonata | | | Coenagrionidae | *Enallagma cyatherigerum* | Northern Bluet | Enallacy | 0 | 0 | 1 | 0 |
| Arthropoda | Insecta | Odonata | | | Coenagrionidae | *Enallagma* sp. | a bluet (odonate) | Enalla_s | 1 | 0 | 0 | 0 |
| Arthropoda | Insecta | Odonata | | | Lestidae | *Lestes congener* | Spotted Spreadwing | Lestesco | 1 | 1 | 1 | 1 |
| Arthropoda | Insecta | Odonata | | | Lestidae | *Lestes disjunctus* | Northern Spreadwing | Lestesdi | 1 | 1 | 1 | 1 |
| Arthropoda | Insecta | Odonata | | | Lestidae | *Lestes unguiculatus* | Lyre-tipped Spreadwing | Lestesun | 1 | 1 | 1 | 1 |
| Arthropoda | Insecta | Odonata | | | Libellulidae | *Sympetrum corruptum* | Variegated Meadowhawk | Sympcor | 1 | 0 | 0 | 0 |
| Arthropoda | Insecta | Odonata | | | Libellulidae | *Sympetrum danae* | Black Meadowhawk | Sympdan | 1 | 1 | 1 | 1 |
| Arthropoda | Ostracoda | Podocopida | | | Candonidae | *Cypria sp.* | an ostracod | Cypria_s | 0 | 0 | 0 | 1 |
| Arthropoda | Insecta | Trichoptera | | | Limnephilidae | *Limnephilus* sp. | a limnephilid caddisfly | Limnep_s | 1 | 1 | 1 | 1 |
| Arthropoda | Insecta | Trichoptera | | | Phryganeidae | *Phryganeid* sp. | a caddisfly | Phryg_o | 1 | 0 | 0 | 0 |
| Arthropoda | Arachnida | Trombidiformes | | | Hydrodromidae | *Hydrodroma sp.* | a water mite | Hydrod_s | 1 | 0 | 1 | 1 |
| Arthropoda | Malacostraca | Amphipoda | | | Gammaridae | *Gammarus lacustris* | an amphipod | Gammala | 0 | 1 | 1 | 0 |
| Arthropoda | Malacostraca | Amphipoda | | | Hyalellidae | *Hyalella "azteca"* | a sideswimmer | Hyalazt | 1 | 1 | 1 | 1 |
| Arthropoda | Maxillopoda | Calanoida | | | Diaptomidae | *Aglaodiaptomus (Diaptomus) forbesi* | a calanoid copepod | Diaptofo | 0 | 1 | 0 | 0 |
| Arthropoda | Maxillopoda | Calanoida | | | Diaptomidae | *Aglaodiaptomus (Diaptomus) leptopus* | a calanoid copepod | Diaptlep | 1 | 1 | 1 | 1 |
| Arthropoda | Maxillopoda | Calanoida | | | Diaptomidae | *Hesperodiaptomus (Diaptomus) arcticus* | a calanoid copepod | Diaptarc | 1 | 1 | 0 | 0 |
| Arthropoda | Maxillopoda | Calanoida | | | Diaptomidae | *Leptodiaptomus (Diaptomus) nudus* | a calanoid copepod | Diaptnu | 1 | 1 | 0 | 1 |
| Arthropoda | Maxillopoda | Calanoida | | | Diaptomidae | *Leptodiaptomus (Diaptomus) sicilis* | a calanoid copepod | Diaptsci | 1 | 0 | 0 | 0 |
| Arthropoda | Maxillopoda | Calanoida | | | Diaptomidae | *Onychodiaptomus (Diaptomus) sanguineus* | a calanoid copepod | Diaptosa | 0 | 1 | 1 | 1 |
| Arthropoda | Maxillopoda | Calanoida | | | Temoridae | Calanoid copepod | Calanoid copepod | Calanoid | 0 | 1 | 1 | 1 |
| Arthropoda | Maxillopoda | Calanoida | | | Temoridae | Calanoid nauplii | Calanoid nauplii | Calannau | 0 | 0 | 0 | 1 |
| Arthropoda | Maxillopoda | Cyclopoida | | | Cyclopidae | Cyclopoid sp. | a cyclopoid copepod | Cyclop_O | 1 | 1 | 1 | 1 |
| Arthropoda | Ostracoda | Podocopida | | | Candonidae | *Candona* sp. | an ostracod | Candon_s | 1 | 0 | 1 | 1 |
| Arthropoda | Ostracoda | Podocopida | | | Cyclocyprididae | *Cypridopsis sp.* | an ostracod | Cyprid_s | 1 | 1 | 1 | 1 |
| Arthropoda | Ostracoda | Podocopida | | | Cyprididae | *Cyclocypris sp.* | an ostracod | Cycloc_s | 0 | 1 | 1 | 1 |
| Arthropoda | Ostracoda | Podocopida | | | Cyprididae | *Cypris pubera* | an ostracod | Cyprispu | 1 | 1 | 1 | 1 |
| Arthropoda | Ostracoda | Podocopida | | | Cyprididae | *Herpetocypris* sp. | an ostracod | Herpet_s | 0 | 0 | 1 | 0 |
| Arthropoda | Ostracoda | Podocopida | | | Cyprididae | *Megalocypris alba* | an ostracod | Megaloal | 1 | 1 | 1 | 1 |
| Arthropoda | Maxillopoda (Copepoda) | |  | Harpacticoida | Harpacticoida fam. | Harpacticoida sp. | a copepod species | Harpact_o | 0 | 1 | 0 | 1 |
| Cnidaria | Hydrozoa | Anthoathecata | | | Hydridae | *Hydra sp.* | freshwater polyp | Hydra_s | 1 | 1 | 0 | 0 |
| Mollusca | Bivalvia | Venerida | | | Sphaeriidae | *Pisidium* sp. | a peaclam | Pisidi_s | 0 | 0 | 1 | 0 |
| Mollusca | Bivalvia | Venerida | | | Sphaeriidae | *Sphaerium* sp. | a fingerclam | Sphaer_s | 0 | 0 | 1 | 1 |
| Arthropoda | Branchiopoda | Diplostraca | | | Chydoridae | *Pleuroxus procurvatus* | a cladoceran crustacean | Pleuropr | 0 | 0 | 1 | 1 |
| Mollusca | Gastropoda | Basommatophora | | | Lymnaeidae | *Stagnicola elodes* | Marsh Pondsnail | Stagelod | 1 | 1 | 1 | 1 |
| Mollusca | Gastropoda | Basommatophora | | | Physidae | *Aplexa elongata ( hypnorum)* | Lance Aplexa | Aplexahy | 0 | 1 | 1 | 1 |
| Mollusca | Gastropoda | Basommatophora | | | Physidae | *Physa jennessi or skinneri* | Obtuse Physa or Glass Physa | Physajk | 1 | 1 | 1 | 1 |
| Mollusca | Gastropoda | Basommatophora | | | Planorbidae | *Gyraulus (Armiger) crista* | Star Gyro | Armigerc | 1 | 1 | 1 | 1 |
| Mollusca | Gastropoda | Basommatophora | | | Planorbidae | *Gyraulus sp.* | a gyro species | Gyraul_s | 1 | 1 | 1 | 1 |
| Mollusca | Gastropoda | Basommatophora | | | Planorbidae | *Planorbella subcrenata (Helisoma trivolvis subcrenatum)* | Rough Ramshorn | Helitrsu | 1 | 1 | 1 | 1 |
| Mollusca | Gastropoda | Basommatophora | | | Planorbidae | *Planorbula campestris* | Meadow Ramshorn | Planorca | 1 | 1 | 1 | 1 |
| Mollusca | Gastropoda | Basommatophora | | | Planorbidae | *Promenetus exacuous exacuous* | Keeled Promenetus | Promexex | 1 | 1 | 1 | 1 |
| Nematoda |  |  | | |  | Nematode | a roundworm | Nematode | 0 | 1 | 1 | 0 |
