# Supplementary material for: Crop cover and nutrient levels mediate the effects of land management type on aquatic invertebrate richness in prairie potholes
Source: PLoS One. 2024 Apr 16;19(4):e0295001. doi: 10.1371/journal.pone.0295001 (PMC11020495; doi:10.1371/journal.pone.0295001)
Supplement: S2 Table — Unexpectedly, levels of nitrogen were highest on organic farms and lowest on conventional sites. However, most forms of phosphorus were highest on minimum tillage farms (except for particulate phosphorus which was highest on organic farms). (DOCX) [file pone.0295001.s002.docx]

| Water Quality | Units | Perennial cover (n=9) | Organic  (n=11) | Minimum tillage (n=11) | Conventional  (n=9) |
| --- | --- | --- | --- | --- | --- |
| Total Dissolved Solids* | 00201L mg/L | 819.11 ± 88.80 | 470.73 ± 166.52 | 417.09 ± 132.76 | 535.33 ± 124.52 |
| Colour true | T 02021L Rel Units | 157.78 ± 28.82 | 153.64 ± 31.14 | 161.36 ± 14.57 | 135.56 ± 17.49 |
| Specific Conductivity | 02041L usie/cm | 1205.33 ± 112.45 | 697.73 ± 202.08 | 644.45 ± 176.73 | 823.44 ± 175.18 |
| Lab |  |  |  |  |  |
| Turbidity | 02081L NTU | 7.68 ± 3.79 | 6.38 ± 2.12 | 3.76 ± 0.81 | 3.77 ± 1.27 |
| Total Organic Carbon* | 06002L mg/L | 33.83 ± 3.80 | 30.81 ± 3.53 | 32.24 ± 1.82 | 29.02 ± 3.66 |
| Dissolved Organic carbon | 06104L mg/L | 31.72 ± 2.89 | 28.21 ± 3.25 | 29.93 ± 2.10 | 27.52 ± 3.72 |
| Bicarbonate* | 06201L mg/L | 367.31 ± 37.48 | 215.84 ± 37.22 | 239.12 ± 31.55 | 259.91 ± 42.15 |
| Free Carbon dioxide* | 06401L mg/L | 10.50 ± 1.50 | 14.13 ± 5.07 | 8.71 ± 0.77 | 7.77 ± 0.98 |
| Particulate Organic Carbon | 06901L mg/L | 2.11 ± 1.14 | 2.60 ± 0.52 | 2.31 ± 0.70 | 1.49 ± 0.71 |
| Total Nitrogen* | 07603L mg/L | 2.99 ± 0.59 | 3.60 ± 1.03 | 3.07 ± 0.33 | 2.56 ± 0.31 |
| Dissolved Nitrogen | 07657L mg/L | 2.65 ± 0.44 | 3.07 ± 0.96 | 2.74 ± 0.35 | 2.33 ± 0.32 |
| Particulate Nitrogen | 07901L mg/L | 0.34 ± 0.17 | 0.37 ± 0.08 | 0.33 ± 0.08 | 0.24 ± 0.10 |
| Fluoride Dissolved | 09117L mg/L | 0.11 ± 0.01 | 0.08 ± 0.01 | 0.15 ± 0.04 | 0.13 ± 0.02 |
| Alkalinity  Total | 10111L mg/L | 301.33 ± 30.75 | 177.06 ± 30.53 | 196.15 ± 25.88 | 213.21 ± 34.58 |
| pH Lab | 10301L pH units | 7.76 ± 0.07 | 7.48 ± 0.11 | 7.61 ± 0.05 | 7.70 ± 0.06 |
| Hardness Total* | 10602L mg/L | 583.14 ± 56.55 | 345.29 ± 121.08 | 266.62 ± 62.43 | 367.19 ± 88.55 |
| Hardness  Non carbonate | 10650L mg/L | 281.81 ± 56.59 | 172.54 ± 93.18 | 74.82 ± 38.35 | 156.19 ± 55.47 |
| Sodium  Percent* | 11250L % | 10.63 ± 2.04 | 6.14 ± 2.06 | 6.95 ± 2.95 | 12.06 ± 4.39 |
| Magnesium Dissolved | 12102L mg/L | 90.38 ± 12.09 | 51.70 ± 21.35 | 33.67 ± 9.90 | 50.15 ± 14.17 |
| Silica  Dissolved | 14108L mg/L | 24.82 ± 8.19 | 8.49 ± 1.97 | 13.16 ± 2.39 | 10.87 ± 3.05 |
| Phosphorus Dissolved Ortho | 15265L mg/L | 0.17 ± 0.07 | 0.83 ± 0.44 | 1.10 ± 0.25 | 0.75 ± 0.14 |
| Phosphorus Total | 15423L mg/L | 0.32 ± 0.08 | 1.09 ± 0.50 | 1.33 ± 0.28 | 0.96 ± 0.16 |
| Phosphorus Dissolved | 15465L mg/L | 0.24 ± 0.08 | 0.97 ± 0.48 | 1.24 ± 0.26 | 0.88 ± 0.16 |
| Phosphorus Particulate | 15901L mg/L | 0.08 ± 0.03 | 0.12 ± 0.04 | 0.09 ± 0 03 | 0.07 ± 0.03 |
| Sulphate Dissolved | 16306L mg/L | 352.43 ± 72.28 | 212.76 ± 105.50 | 132.25 ± 71.56 | 204.21 ± 66.56 |
| Chloride Dissolved | 17206L mg/L | 10.26 ± 1.28 | 5.64 ± 1.28 | 11.13 ± 6.11 | 21.15 ± 10.57 |
| Calcium Dissolved | 20103L mg/L | 84.52 ± 7.31 | 53.03 ± 13.55 | 51.26 ± 9.13 | 64.37 ± 12.95 |
| Potassium Dissolved | 100499 mg/L | 37.71 ± 4.63 | 20.88 ± 1.47 | 29.06 ± 2.92 | 25.29 ± 4.07 |
| Sodium Dissolved | 11103L mg/L | 38.13 ± 10.20 | 22.60 ± 9.67 | 28.66 ± 20.37 | 31.24 ± 12.85 |
