# Supplementary material for: Crop cover and nutrient levels mediate the effects of land management type on aquatic invertebrate richness in prairie potholes
Source: PLoS One. 2024 Apr 16;19(4):e0295001. doi: 10.1371/journal.pone.0295001 (PMC11020495; doi:10.1371/journal.pone.0295001)
Supplement: S3 Table — Although cropland cover was highest on conventional farms (and about twice that on perennial cover sites), these farms also had the highest average cover of tall shrubs (which intercept runoff from snowmelt). (DOCX) [file pone.0295001.s003.docx]

|  | Perennial cover | | Organic | | Minimum tillage | | Conventional | |
| --- | --- | --- | --- | --- | --- | --- | --- | --- |
|  | Mean | SE | Mean | SE | Mean | SE | Mean | SE |
| Cropland | 155.54 | 25.90 | 230.65 | 20.59 | 253.92 | 10.29 | 266.52 | 9.14 |
| Hay crop | 1.16 | 1.16 | 11.00 | 5.82 | 1.17 | 1.06 | 0.76 | 0.76 |
| Native dominant grassland | 99.77 | 28.09 | 25.66 | 9.27 | 25.21 | 6.94 | 10.10 | 5.21 |
| Tall shrubs | 6.22 | 3.68 | 4.78 | 2.63 | 1.95 | 1.04 | 7.12 | 5.23 |
| Pasture | 0 | 0 | 0 | 0 | 0 | 0 | 1.49 | 1.49 |
| Hardwood (open canopy) | 19.35 | 7.22 | 14.67 | 6.32 | 8.15 | 4.05 | 8.07 | 4.37 |
| Hardwood (closed canopy) | 0.32 | 0.32 | 1.53 | 1.38 | 0 | 0 | 0.63 | 0.52 |
| Jack pine (closed canopy) | 0 | 0 | 0.31 | 0.28 | 0 | 0 | 0 | 0 |
| Jack pine (open canopy) | 0 | 0 | 0.44 | 0.40 | 0 | 0 | 0 | 0 |
| Spruce (closed canopy) | 0 | 0 | 0.74 | 0.67 | 0 | 0 | 0 | 0 |
| Spruce (open canopy) | 0 | 0 | 0.13 | 0.12 | 0 | 0 | 0 | 0 |
| Mixed woods | 0 | 0 | 1.33 | 1.20 | 0 | 0 | 0 | 0 |
| Open water | 12.16 | 4.20 | 4.75 | 3.31 | 4.21 | 1.56 | 8.33 | 3.24 |
| Marsh | 14.70 | 5.83 | 7.66 | 1.97 | 10.87 | 2.01 | 9.39 | 1.71 |
| Mud/sand/saline | 4.39 | 2.55 | 1.46 | 1.18 | 0.89 | 0.81 | 0.32 | 0.22 |
| Total water^1^ | 31.25 | 7.49 | 13.86 | 4.15 | 15.97 | 3.57 | 18.04 | 3.53 |
| Farmstead and urban | 0.49 | 0.38 | 1.97 | 0.60 | 1.61 | 0.58 | 1.60 | 1.13 |

^1^ Total water is ‘water bodies’ plus ‘Marsh’, and Mud/sand/saline combined.
