# Supplementary material for: Crop cover and nutrient levels mediate the effects of land management type on aquatic invertebrate richness in prairie potholes
Source: PLoS One. 2024 Apr 16;19(4):e0295001. doi: 10.1371/journal.pone.0295001 (PMC11020495; doi:10.1371/journal.pone.0295001)
Supplement: S4 Table — Also shown are the null probabilities (p values) used to calculate Fisher’s C statistic to test the correlational structure of the full path model in Fig 1. The independence claim notation identifies the tested pair of variables in parentheses, followed by the variables that were statistically controlled for while testing the independence between the pair, in curled brackets. Two arrows were added to the path diagram (between crop and management and nutrients and turbidity) following the independence test based on this set of claims. With these arrows added, the p-value of the Fisher’s C statistic for the path model was 0.74. (DOCX) [file pone.0295001.s004.docx]

| Independence claim | Model ^a^ | *p* value |
| --- | --- | --- |
| (CROP, LM-TYPE) | CROP ~ LM-TYPE | 0.0005 |
| (WET, LM-TYPE) | WET ~ LM-TYPE | 0.0957 |
| (CROP, WET) | CROP ~ WET | 0.2512 |
| (TNU, TUR) {LM-TYPE, CROP, WET} | TNU ~ TUR + LM-TYPE + CROP + WET | < 0.0001 |

*Notes:* LM-TYPE = land management type, CROP = cropland cover, WET = wetland cover, TNU = total nutrients, TUR = turbidity. ^a^ Relationships were tested using linear mixed models with farm cluster as a random factor.
