# Supplementary material for: Crop cover and nutrient levels mediate the effects of land management type on aquatic invertebrate richness in prairie potholes
Source: PLoS One. 2024 Apr 16;19(4):e0295001. doi: 10.1371/journal.pone.0295001 (PMC11020495; doi:10.1371/journal.pone.0295001)
Supplement: S5 Table — Relationships were modeled by linear mixed models with cluster as a random factor. Presented are the Akaike Information Criterion (AIC) and change in AIC (relative to the most supported model; ΔAIC) for all models with ΔAIC ≤ 2. (DOCX) [file pone.0295001.s005.docx]

| Cropland cover as a function of | Nutrient level as a function of | Turbidity as a function of | Species richness as a function of | AIC | ∆AIC |
| --- | --- | --- | --- | --- | --- |
| land management | cropland + turbidity | cropland | nutrient level | 49.824 | 0.000 |
| land management | cropland + turbidity | cropland | cropland + nutrient level | 50.175 | 0.351 |
| land management | cropland + turbidity | land management | nutrient level | 50.434 | 0.610 |
| land management | cropland + turbidity | land management | cropland + nutrient level | 50.687 | 0.863 |
| land management | turbidity | cropland | nutrient level | 50.832 | 1.008 |
| land management | cropland + turbidity | cropland | nutrient level + turbidity | 51.175 | 1.351 |
| land management | turbidity | cropland | cropland + nutrient level | 51.183 | 1.359 |
| land management | cropland + land management + turbidity | cropland | nutrient level | 51.267 | 1.443 |
| land management | turbidity | land management | nutrient level | 51.443 | 1.619 |
| land management | cropland + land management + turbidity | cropland | cropland + nutrient level | 51.618 | 1.794 |
| land management | turbidity | land management | cropland + nutrient level | 51.696 | 1.872 |
| land management | cropland + turbidity | cropland + land management | nutrient level | 51.752 | 1.928 |
| land management | cropland + turbidity | cropland | cropland + nutrient level + turbidity | 51.787 | 1.963 |
