# Supplementary material for: Crop cover and nutrient levels mediate the effects of land management type on aquatic invertebrate richness in prairie potholes
Source: PLoS One. 2024 Apr 16;19(4):e0295001. doi: 10.1371/journal.pone.0295001 (PMC11020495; doi:10.1371/journal.pone.0295001)
Supplement: S6 Table — The p-value of the Fisher’s C statistic for the path model was 0.961. (DOCX) [file pone.0295001.s006.docx]

| Response | Predictor | Effect | SE | Critical value | P | Marginal R^2^ | Conditional R^2^ |
| --- | --- | --- | --- | --- | --- | --- | --- |
| cropland | organic farming | 1.048 | 0.336 | 3.115 | 0.0044 | 0.36 | 0.50 |
|  | minimum till | 1.378 | 0.336 | 4.097 | 0.0004 |  |  |
|  | conventional | 1.607 | 0.356 | 4.514 | 0.0001 |  |  |
| nutrient levels | cropland cover | 0.257 | 0.119 | 2.163 | 0.0396 | 0.49 | 0.49 |
|  | turbidity | 0.672 | 0.119 | 5.664 | <0.001 |  |  |
| turbidity | cropland cover | -0.094 | 0.162 | -0.581 | 0.5662 | 0.01 | 0.01 |
| richness | nutrient levels | -3.463 | 1.580 | -2.192 | 0.0369 | 0.11 | 0.11 |
