# Supplementary material for: Crop cover and nutrient levels mediate the effects of land management type on aquatic invertebrate richness in prairie potholes
Source: PLoS One. 2024 Apr 16;19(4):e0295001. doi: 10.1371/journal.pone.0295001 (PMC11020495; doi:10.1371/journal.pone.0295001)
Supplement: S7 Table — (DOCX) [file pone.0295001.s007.docx]

| Predictor | Effect | SE | Critical value | P |
| --- | --- | --- | --- | --- |
| organic farming | 0.250 | 4.478 | 0.056 | 0.956 |
| minimum tillage | 2.886 | 4.478 | 0.645 | 0.525 |
| conventional | 3.014 | 4.730 | 0.637 | 0.530 |
