# Supplementary material for: Crop cover and nutrient levels mediate the effects of land management type on aquatic invertebrate richness in prairie potholes
Source: PLoS One. 2024 Apr 16;19(4):e0295001. doi: 10.1371/journal.pone.0295001 (PMC11020495; doi:10.1371/journal.pone.0295001)
Supplement: S8 Table — Significance ** P < 0.01; * P < 0.05. (DOCX) [file pone.0295001.s008.docx]

| **Source** | **Df** | **SS** | **F** | **P** |
| --- | --- | --- | --- | --- |
| Cropland | 1 | 0.319 | 2.778 | 0.0045 ** |
| Wetland | 1 | 0.255 | 2.224 | 0.0237 * |
| Nutrient levels | 1 | 0.051 | 0.441 | 0.8679 |
| Turbidity | 1 | 0.169 | 1.473 | 0.1816 |
| Land management type | 3 | 0.569 | 1.654 | 0.0337 * |
| Residuals | 32 | 3.670 |  |  |
