# Supplementary figures and images for: Crop cover and nutrient levels mediate the effects of land management type on aquatic invertebrate richness in prairie potholes
Source: PLoS One. 2024 Apr 16;19(4):e0295001. doi: 10.1371/journal.pone.0295001 (PMC11020495; doi:10.1371/journal.pone.0295001)

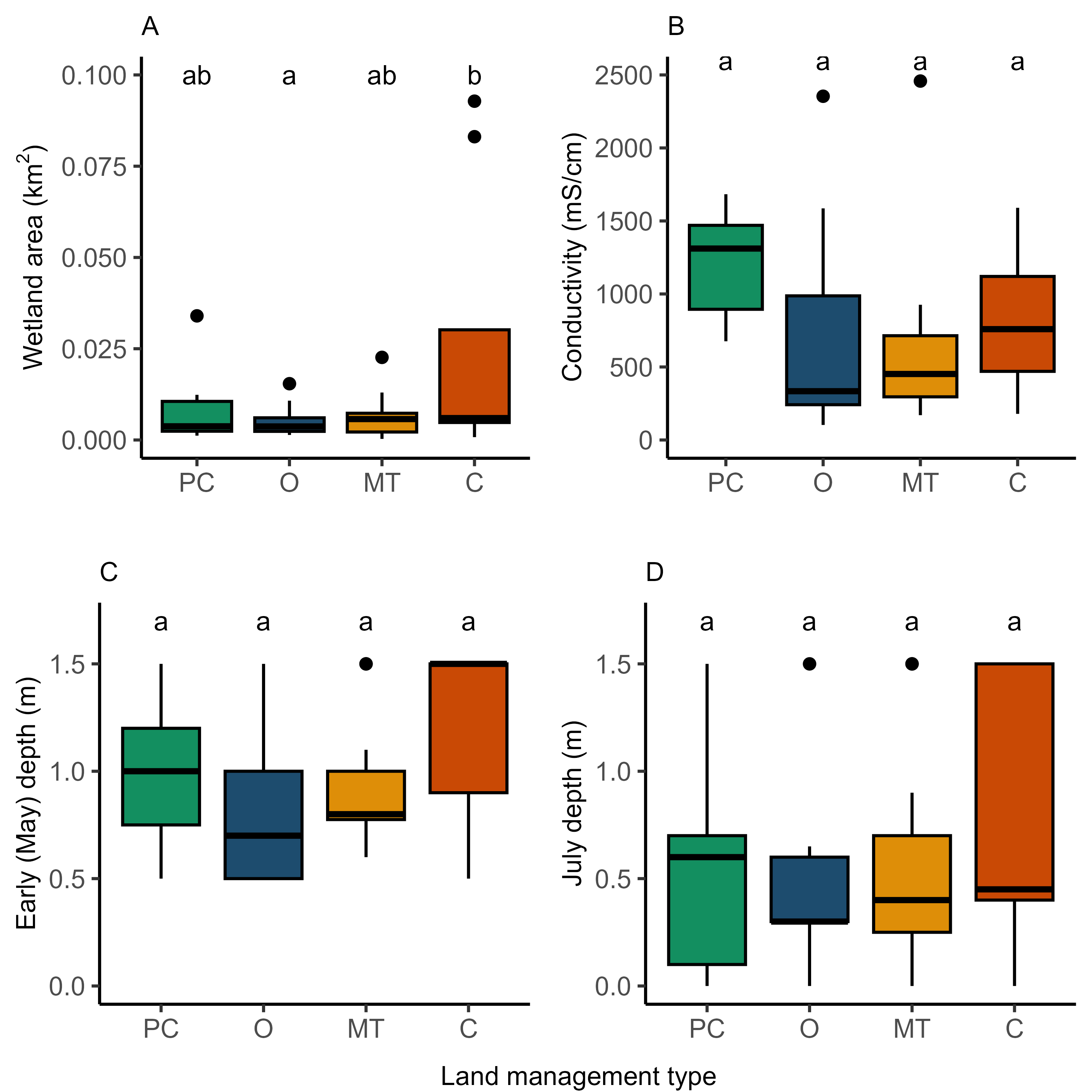

Supplement: S1 Fig — PC = perennial cover; O = organic; MT = minimum tillage; C = conventional. Box and whisker plots summarizing median, inter-quantile range, 1.5 times the inter-quartile range, and extreme values. The population means of land management types with the same letter are not significantly different at alpha = 0.05 (Tukey HSD method). (TIFF) [file pone.0295001.s009.tiff]

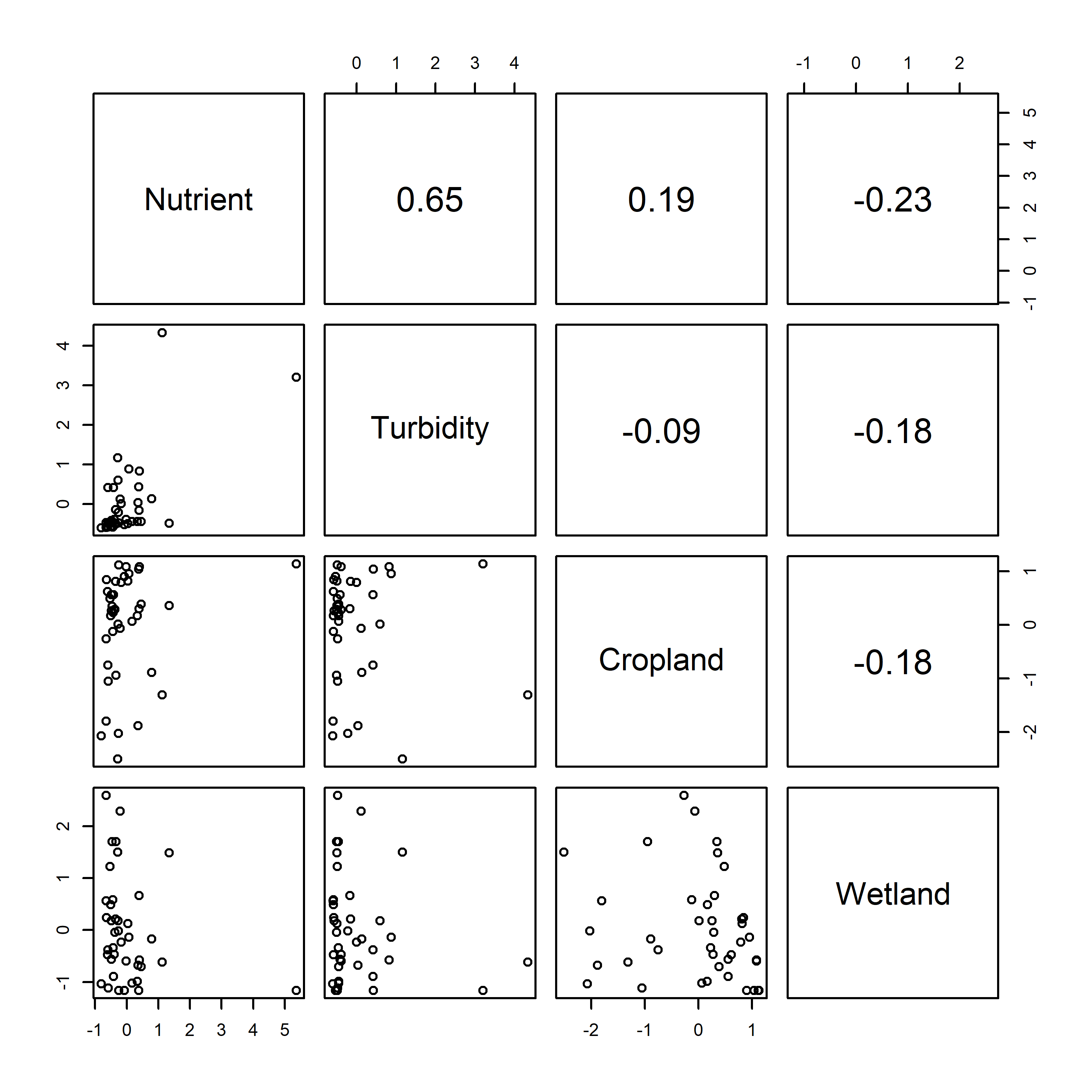

Supplement: S2 Fig — (TIFF) [file pone.0295001.s010.tiff]

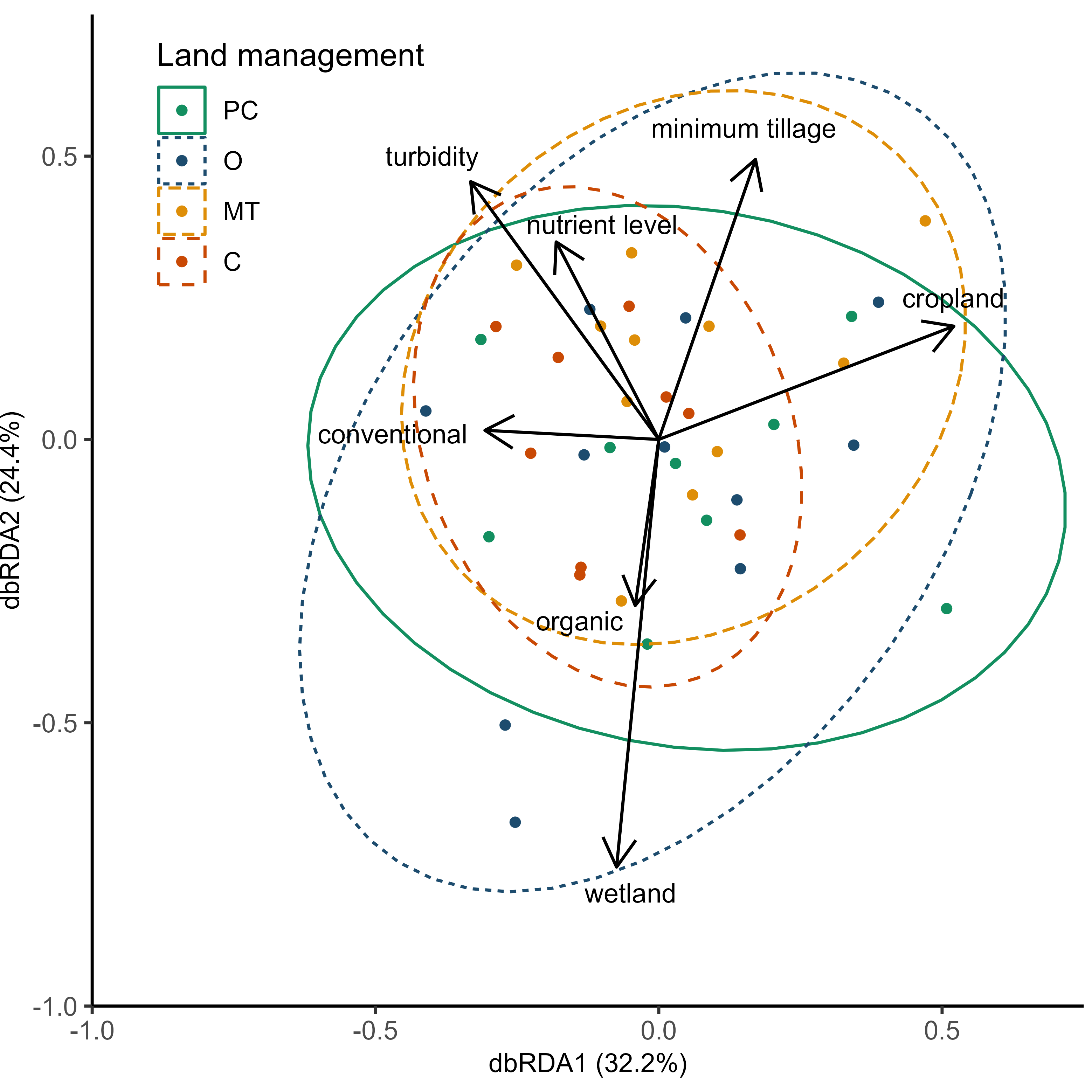

Supplement: S3 Fig — Ellipses show 95% contours for land management types. The vectors show the direction and strength of relationship (length) in the ordination. Land management types: PC = perennial cover; O = organic; MT = minimum tillage; C = conventional. (TIFF) [file pone.0295001.s011.tiff]

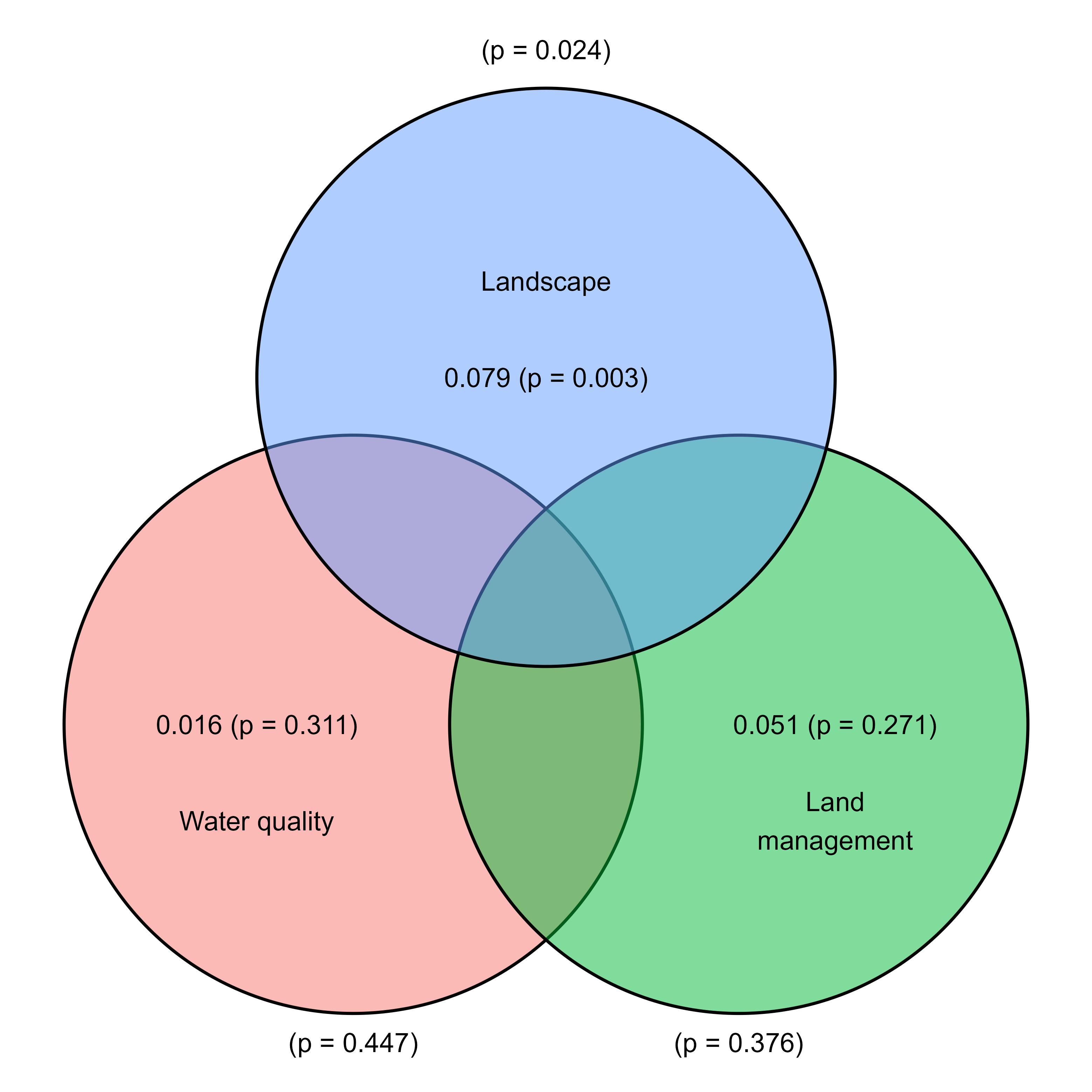

Supplement: S4 Fig — Diagram shows beta-diversity explained by land management type, landscape and local covariates across 40 sites (not including space). Values within ellipses are the conditional effects after controlling for other covariates (% variation explained and associated p-value). P-values outside ellipses for each covariate are the significances of the marginal effects. (TIFF) [file pone.0295001.s012.tiff]

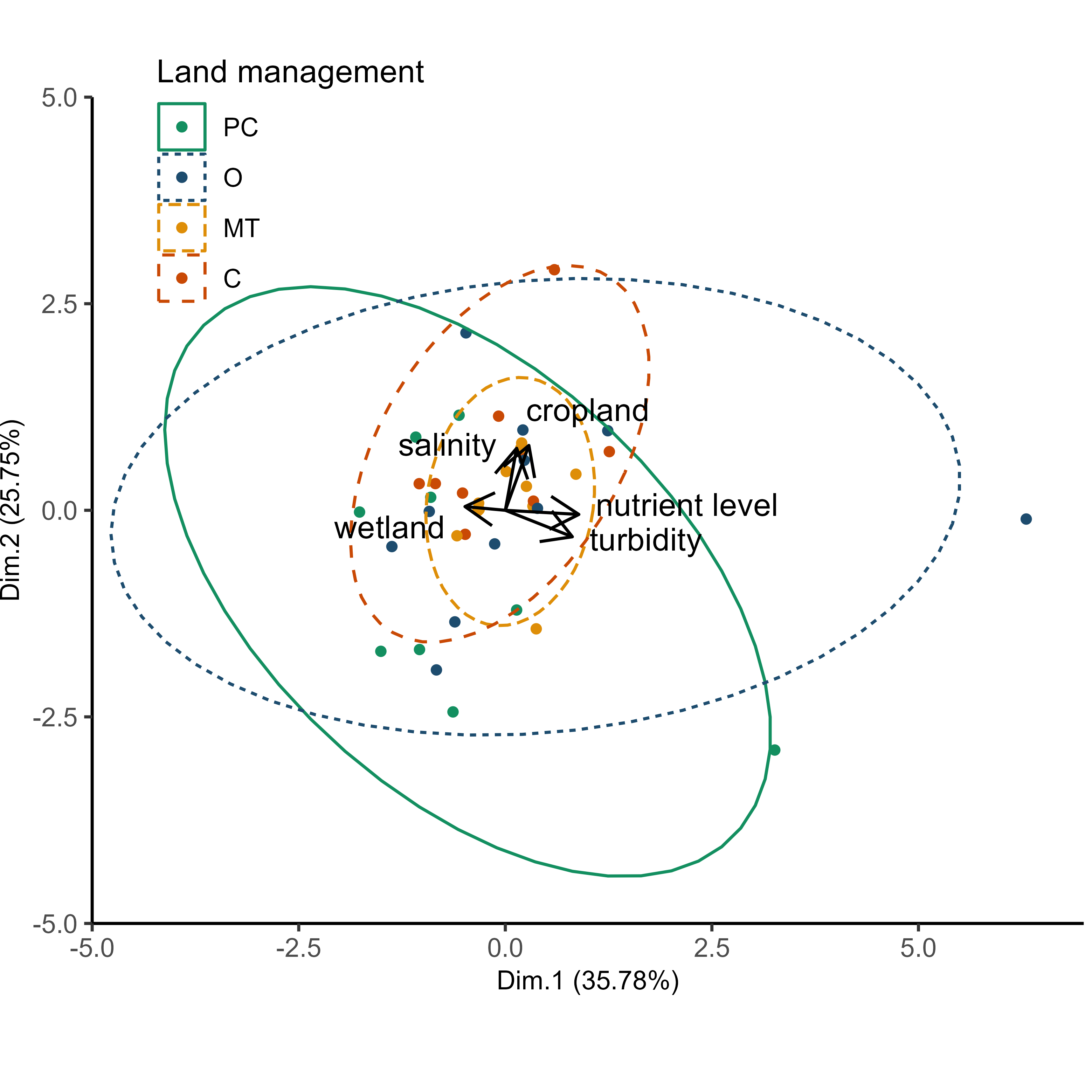

Supplement: S5 Fig — Salinity was derived from Surficial geology of Saskatchewan soil maps (Government of Canada 2023: Agri-Environmental Indicator–Risk of Soil Salinization. https://open.canada.ca/data/en/dataset/8e2583c8-af71-4873-8cca-8081db789121 and https://agriculture.canada.ca/en/agricultural-production/soil-and-land/soil-salinization-indicator). Land management: PC = perennial cover; O = organic; MT = minimum tillage; C = conventional). Note that salinity was correlated with cropland cover but salinity levels did not approach toxicity levels for invertebrates (K. McLean, USGS, pers. comm.). (TIFF) [file pone.0295001.s013.tiff]

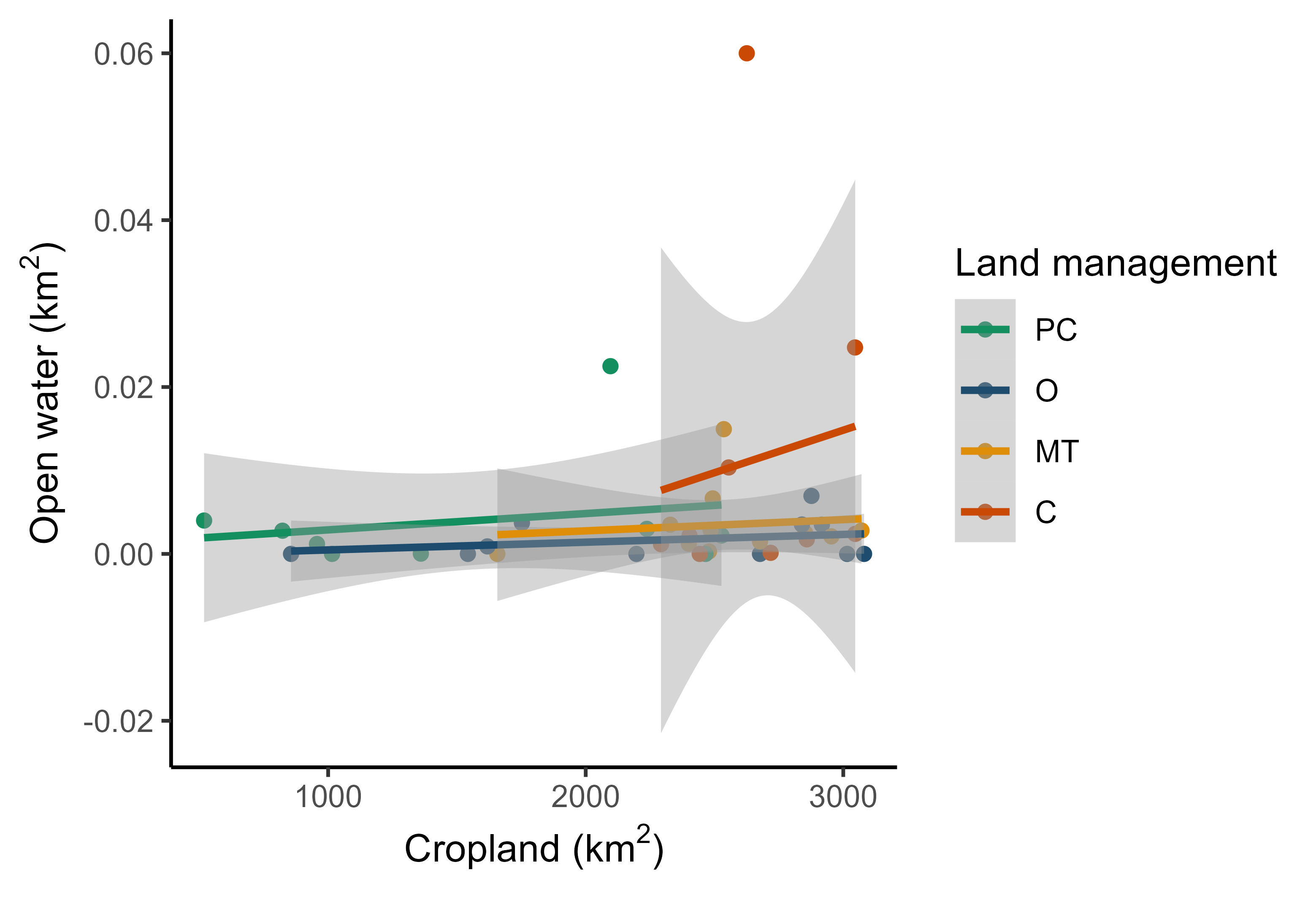

Supplement: S6 Fig — (TIFF) [file pone.0295001.s014.tiff]

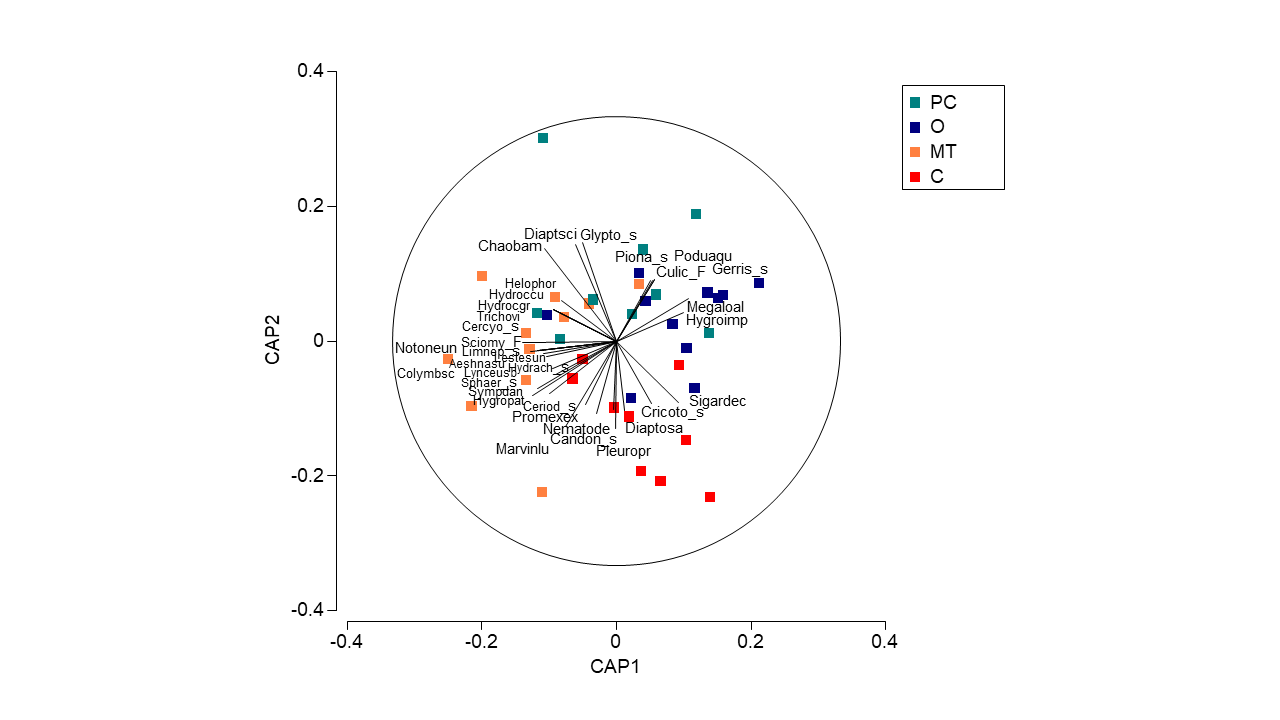

Supplement: S7 Fig — Some separation of land management types occurred on axes 1 and 2, with organic sites on the right side of the ordination and conventional sites on the left, but there was no significant difference among farm types (P > 0.1). The CAP analysis indicated that there was a high misclassification error in assigning wetlands to farm management types (30% correct, 70% misclassified). Minimum tillage had the highest percentage of correct classifications (45.5%). Many species appeared to be associated with these sites (e.g., Notonecta undulata, Aeshna subarctica, Colymbetes sculptilis; Pearson correlation 0. 3). See S1 Table for species abbreviations. (TIFF) [file pone.0295001.s015.tiff]
