# Supplementary material for: Crop cover and nutrient levels mediate the effects of land management type on aquatic invertebrate richness in prairie potholes
Source: PLoS One. 2024 Apr 16;19(4):e0295001. doi: 10.1371/journal.pone.0295001 (PMC11020495; doi:10.1371/journal.pone.0295001)
Supplement: S1 Appendix — (DOCX) [file pone.0295001.s016.docx]

**Additional Discussion**

*Multivariate analyses*

Land management type was not significant when analyzed for corrected dissimilarities for the test for the homogeneity of multivariate dispersions. However, beta diversity followed a trend from highest values on low intensity land management types (i.e., organic farms) to lowest values on high intensity land management types (i.e., conventional farms). We speculate that the suggested higher beta diversity on organic farms and perennial cover sites was because wetlands were smaller and shallower on these land management types. On organic sites, this may also be due to the greater diversity of native land cover features (e.g., closed canopy hardwood forests) compared to other management types, even though in the prairies organic farmers often cultivate to the edge of wetland margins[1]. One interesting finding was that in the case of covariates, some organic farm sites were outliers (see S5 Fig) and this may have partly accounted for their higher beta diversity due to the wider range of environmental conditions they experienced.

In the PPR, conservation efforts for waterfowl and other elements of biodiversity have often been directed towards landscapes containing high densities of wetlands (small, ephemeral and temporary) embedded within a grassland matrix [2]. However, when grasslands are converted to row crop production, landscape characteristics are altered, and hydrology is impacted because many small wetlands tend to become amalgamated to form fewer, larger wetlands [3,4]. While this may not affect overall species richness, it can have a negative influence on beta diversity because a key component of differentiation in aquatic invertebrate communities is wetland hydroperiod. Shifts in hydroperiod caused by row crop agriculture can lead to a decline in beta diversity because smaller drained wetlands or more generally those with short hydroperiods often have unique communities which disproportionately contribute to beta diversity [5–7].

*Path model*

It should be emphasized that our path model controlled for landscape factors such as cropland amount and wetland area, as well as within-pond chemistry (nutrients and turbidity), so the differences in the direct effects of land management types on richness must be due to other factors. We speculate that there was some other aspect of organic farm management not accounted for in our models that diminished richness of aquatic invertebrates relative to the other three land management types.

Although the direct effect of land management type on aquatic invertebrate richness was not supported, we note that the coefficients for this relationship were not consistent with our expectation that perennial cover and organic farming would be associated with greater species richness than either minimum tillage or conventional farming, because of differences in synthetic pesticide use. Instead, we found similar direct effects of perennial cover and conventional farming on aquatic invertebrate richness, whereas relative to perennial cover, organic land management had a more negative direct effect, and minimum tillage a more positive direct effect on richness relative to perennial cover (the reference category). Interestingly, the latter finding is consistent with what has been observed in streams in Ontario, where streams adjacent to conservation tillage systems held significantly more taxa overall—specifically, more species of Ephemeroptera, Plecoptera and Trichoptera—than streams beside conventionally-plowed land [8]

First, it is possible that the negative effect of organic farming on aquatic invertebrates relative to the other three land management types was due to the increased tillage used by organic farmers for weed control, or other disturbance, such as haying. Supporting the speculation regarding disturbance, organic farms had almost 10 times the amount of hay cover in the landscapes surrounding wetlands than other land management types (S3 Table**)**. That disturbance on the land surrounding wetlands may be a factor in the lower species richness on organic farms is also supported by the finding that minimum tillage land management was associated with higher species richness than the other three management types.

We also speculated that the negative effect of organic farming on aquatic invertebrates relative to the other three land management types could result from differences in the width of vegetation buffers around wetlands. Organic farmers in the region were known to often cultivate to the edge of wetlands which could result in loss of vegetation structure [1]. The width of vegetation buffers around wetlands can play an important role in mitigating run-off from surrounding lands and consequently impact aquatic invertebrate communities [9]. For example, Wade [10] recently found that wider vegetation buffers between cropland and wetland areas in Saskatchewan increased richness of aquatic invertebrates [86]. Although buffer width was not measured in our study, data collected for another part of the project at the same wetlands [11] did not indicate that wetlands embedded in organic farmland had less willow and other shrub cover around their margins than other land management types; in fact, they tended to have more. This suggests that differences in wetland buffers among land management types are unlikely to explain our findings. Also, most wetlands selected had minimal direct disturbance at their edges. Outside influences could also affect water quality in ponds on organic farms and have repercussions on aquatic invertebrate abundance and richness; interestingly, Bates and Hall [12] found higher levels of methyl-mercury (MeHg) in invertebrates from ponds surrounded by organic cultivation than those surrounded by grassland or traditional agriculture. They attributed this to atmospheric sources or higher uptake of the aquatic invertebrates in ponds embedded in organic farmland.

We also investigated whether our findings could be explained by confounding relationships between land management type and wetland size, conductivity, or water depth. We did find a relationship between land management type and wetland size, with the land management type having the lowest aquatic invertebrate species richness (organic farms) tending to have the smallest wetlands (S1 Fig). Thus, it is possible that the smaller wetlands on organic farms had shorter hydroperiods and reduced spatial connectivity. The ratio of open water to vegetated area, and the overall amount of water related to hydroperiod are important drivers for aquatic invertebrate communities, such as in small fishless wetlands in North Dakota [5,7], prairie wetlands in Alberta [13] and globally [14]. However, differences in wetland size cannot fully explain the observed direct relationship between land management type and aquatic invertebrate richness because, compared to perennial cover, minimum tillage management had the greatest positive direct effect on aquatic invertebrates, despite having wetlands that were not significantly different in size to those on conventional, perennial cover, or organic sites (S1 Fig).

It is possible that different land management types are likely to be found in areas with different geological or soil conditions, and that those conditions influenced aquatic invertebrate richness, Areas with high potential for crop production (conventional and minimum tillage sites) are most likely to be located in areas with the most fertile soils and gentle topography. By contrast—as is often the case for other land restoration programs—perennial cover sites, including those from the PCP, were relegated to areas with less potential for crop production and thus potentially had lower wetland productivity [15]. However, we think this is an unlikely explanation for our findings, because our study was specifically designed to address this possibility, by locating land management types in clusters. Additionally, if this was the case, we would have expected perennial cover sites to have lower aquatic invertebrate species richness than conventional ones, whereas in fact they were similar.

Another possible, but unlikely, explanation for the observed direct effect of land management type on aquatic invertebrate richness is the presence/absence of fish (or other predators) in sampled wetlands. Although most Prairie Pothole wetlands are fishless, fish predation can mask relationships (i.e., effect sizes detected in statistical models) between aquatic invertebrates and water chemistry, as well as apparent responses to surrounding landscapes [7]. Fish were only detected at one wetland during the sampling period, but they could nevertheless have possibly colonized wetlands during flooding events; a few individual fish could exert a disproportionate impact on invertebrate community structure. While historically fish were largely absent from prairie potholes due to episodic drying events, the shallowness of most wetlands, winter freezes and high salinity [16], with increased precipitation as a result of climate change this situation may change and fish are colonizing many water bodies [17]. Other predators could include tiger salamanders (*Ambystoma* spp.; [18]), Northern Leopard Frogs (*Lithobates pipiens*) and various predatory invertebrates. In addition, waterfowl may have exerted some predation pressure but there is no reason to suspect that fish were more likely to occur in wetlands on organic sites or less likely to occur in minimum tillage sites than the other land management types.

Finally, there is a possibility that some sampling biases related to pond water depth could affect our results; for example, sweep nets may not function as effectively in vegetation as in open water and water depth can influence sampling efficiency (e.g., for funnel traps). However, it is difficult to envisage how such factors could introduce systematic bias among the different land management types, especially given that wetlands were selected to minimize variation in wetland depth across sites (see Methods).

**References**

1. Clark RG, Boutin C, Jobin B, Forsyth DJ, Shutler D, Leeson JY, et al. Living on the edge: Field boundary habitats, biodiversity and agriculture. In: Thomas AG, editor. Field Boundary Habitats: Implications for Weed, Insect and Disease Management. Sainte-Anne-de Bellevue, Québec: Canadian Weed Science Society – Société canadienne de malherbologie; 2005. pp. 113–133.

2. Janke AK, Anteau MJ, Stafford JD. Prairie wetlands confer consistent migrant refueling conditions across a gradient of agricultural land use intensities. Biol Conserv. 2019;229: 99–112. https://doi:10.1016/j.biocon.2018.11.021

3. Serran JN, Creed IF. New mapping techniques to estimate the preferential loss of small wetlands on prairie landscapes. Hydrol Process. 2016;30: 396–409. https://doi:10.1002/hyp.10582

4. Van Meter KJ, Basu NB. Signatures of human impact: size distributions and spatial organization of wetlands in the Prairie Pothole landscape. Ecological Applications. 2015;25: 451–465. https://doi:10.1890/14-0662.1

5. McLean KI, Mushet DM, Sweetman JN, Anteau MJ, Wiltermuth MT. Invertebrate communities of Prairie-Pothole wetlands in the age of the aquatic Homogenocene. Hydrobiologia. 2020;847: 3773–3793. https://doi:10.1007/s10750-019-04154-4

6. Schriever TA, Williams DD. Influence of pond hydroperiod, size, and community richness on food-chain length. Freshw Sci. 2013;32: 964–975. https://doi:10.1899/13-008.1

7. McLean KI, Mushet DM, Newton WE, Sweetman JN. Long-term multidecadal data from a prairie-pothole wetland complex reveal controls on aquatic-macroinvertebrate communities. Ecol Indic. 2021;126. https://doi:10.1016/j.ecolind.2021.107678

8. Barton DR, Farmer MED. The effects of conservation tillage practices on benthic invertebrate communities in headwater streams in southwestern Ontario, Canada. Environ Pollut. 1997;96: 207–215. https://doi.org/10.1016/S0269-7491(97)00020-1

9. Prosser RS, Hoekstra PF, Gene S, Truman C, White M, Hanson ML. A review of the effectiveness of vegetated buffers to mitigate pesticide and nutrient transport into surface waters from agricultural areas. J Environ Manage. 2020;261. https://doi.org/10.1016/j.jenvman.2020.110210

10. Wade A. Role of vegetated buffer zones for mitigating wetland pesticide contamination and protecting aquatic invertebrate communities in northern prairie wetlands. M.Sc. Thesis, University of Saskatchewan, Saskatchewan, Canada. 2021.

11. Kirk DA, Martínez-Lanfranco J-A, Forsyth DJ, Martin AE. Farm management and landscape context shapes plant diversity at wetland edges in the prairie pothole region of Canada. Ecol Appl. In press. 2023.

12. Bates LM, Hall BD. Concentrations of methylmercury in invertebrates from wetlands of the Prairie Pothole Region of North America. Environ Pollut. 2012;160: 153–160. https://doi:10.1016/j.envpol.2011.08.040

13. Gleason JE, Rooney RC. Pond permanence is a key determinant of aquatic macroinvertebrate community structure in wetlands. Freshw Biol. 2018;63: 264–277. https://doi:10.1111/fwb.13057

14. Epele LB, Grech MG, Williams-Subiza EA, Stenert C, McLean K, Greig HS, et al. Perils of life on the edge: Climatic threats to global diversity patterns of wetland macroinvertebrates. Sci Total Environ. 2022;820. https://doi:10.1016/j.scitotenv.2022.153052

15. Claassen R, Cattaneo A, Johansson R. Cost-effective design of agri-environmental payment programs: U.S. experience in theory and practice. Ecological Economics. 2008;65: 737–752. https://doi:10.1016/j.ecolecon.2007.07.032

16. Kantrud HA, Miller JB, van der Valk AG. Vegetation of wetlands of the prairie pothole region. In: van der Valk AG, editor. Northern Prairie Wetlands. Ames, Iowa, USA: Iowa State University Press; 1989. pp 137-189.

17. McLean KI, Mushet DM, Stockwell CA. From “Duck Factory” to “Fish Factory”: Climate induced changes in vertebrate communities of prairie pothole wetlands and small lakes. Wetlands. 2016;36: 407–421. https://doi:10.1007/s13157-016-0766-3

18. Benoy GA. Tiger salamanders in prairie potholes: A “Fish in amphibian’s garments?” Wetlands. 2008;28: 464–472. https://doi: 10.1672/07-07.1
